# Comprehensive software suite for functional analysis and synaptic input mapping of dendritic spines imaged *in vivo*

**DOI:** 10.1117/1.NPh.11.2.024307

**Published:** 2024-04-16

**Authors:** Yiyi Yu, Liam M. Adsit, Ikuko T. Smith

**Affiliations:** a University of California, Santa Barbara, Department of Electrical and Computer Engineering, Santa Barbara, California, United States; b University of California, Santa Barbara, Department of Molecular, Cellular and Developmental Biology, Santa Barbara, California, United States; c University of California, Santa Barbara, Neuroscience Research Institute, Santa Barbara, California, United States; d University of California, Santa Barbara, Department of Psychological and Brain Sciences, Santa Barbara, California, United States

**Keywords:** dendrites, spines, input mapping, spine turnover, cross-session alignment, point-cloud registration

## Abstract

**Significance:**

Advances in genetically encoded sensors and two-photon imaging have unlocked functional imaging at the level of single dendritic spines. Synaptic activity can be measured in real time in awake animals. However, tools are needed to facilitate the analysis of the large datasets acquired by the approach. Commonly available software suites for imaging calcium transients in cell bodies are ill-suited for spine imaging as dendritic spines have structural characteristics distinct from those of the cell bodies. We present an automated tuning analysis tool (AUTOTUNE), which provides analysis routines specifically developed for the extraction and analysis of signals from subcellular compartments, including dendritic subregions and spines.

**Aim:**

Although the acquisition of *in vivo* functional synaptic imaging data is increasingly accessible, a hurdle remains in the computation-heavy analyses of the acquired data. The aim of this study is to overcome this barrier by offering a comprehensive software suite with a user-friendly interface for easy access to nonprogrammers.

**Approach:**

We demonstrate the utility and effectiveness of our software with demo analyses of dendritic imaging data acquired from layer 2/3 pyramidal neurons in mouse V1 *in vivo*. A user manual and demo datasets are also provided.

**Results:**

AUTOTUNE provides a robust workflow for analyzing functional imaging data from neuronal dendrites. Features include source image registration, segmentation of regions-of-interest and detection of structural turnover, fluorescence transient extraction and smoothing, subtraction of signals from putative backpropagating action potentials, and stimulus and behavioral parameter response tuning analyses.

**Conclusions:**

AUTOTUNE is open-source and extendable for diverse functional synaptic imaging experiments. The ease of functional characterization of dendritic spine activity provided by our software can accelerate new functional studies that complement decades of morphological studies of dendrites, and further expand our understanding of neural circuits in health and in disease.

## 1 Introduction

*In vivo* functional imaging of neuronal activity has greatly advanced in recent years with the advent of activity indicators, such as genetically encoded calcium indicators (GECIs), glutamate sensors, and genetically encoded voltage indicators (GEVIs^1^^–^^5^). Of these, calcium imaging with the GCaMP series of GECIs has been the most popular.^2^^,^^6^^–^^8^ Although electrophysiological recording of neuronal activity remains a gold standard for assessing neuronal activity, calcium imaging with GECIs has numerous advantages. One can simultaneously measure the activity of a population of neurons and/or from multiple compartments within a single cell. The former has especially been embraced by systems neuroscientists and led to the recent spur of inventions of headmounted miniscopes^9^^–^^13^ and large field-of-view mesoscopes,^14^^–^^17^ with the latter allowing for simultaneous imaging of neurons from different brain regions that are millimeters apart. These versatile approaches can be applied in longitudinal functional imaging of neurons spanning different stages of behavior, learning, and circuit development.

On a single-neuron level, functional mapping of synaptic inputs on dendrites using activity indicators has emerged as a novel and powerful way to probe dendritic mechanisms for synaptic integration.^18^^–^^23^ By characterizing the dendritic spine-specific activity relative to sensory stimulus features (e.g., orientation of gratings, angles of whisker deflection, and pure tone frequency), one can determine the stimulus evoked response properties of the presynaptic inputs impinging onto individual dendritic spines. Such an approach provides physiological insights into the single-neuron computations that are capable of generating reliable yet adaptive output.

Due to its technical demands (e.g., high-speed imaging with custom two-photon microscopes^24^), *in vivo* functional input mapping has traditionally been performed by only a handful of leading experts in the field of synaptic and dendritic physiology. Their studies have delivered insightful findings about circuit connectivity and synaptic integration.^19^^–^^24^ However, recent advances in commercially available two-photon microscopy and GECIs have revolutionized the field such that these technologically demanding experimental approaches are ever more accessible to researchers with a wide range of expertise.

Structural studies of dendritic spine morphologies and changes thereof, including chronic *in vivo* imaging, have provided links to brain functions, such as developmental plasticity, learning and memory, and the hormonal regulation of circuit function.^25^^–^^32^ Dendritic spine structures are also known to be altered in neurodegenerative diseases and cognitive dysfunctions, including Alzheimer’s disease, schizophrenia, autism, and traumatic brain injury.^33^^,^^34^ Functional characterization of dendritic spine activity can complement these morphological studies and offer further mechanistic insights that can expand our understanding of neural circuits in health and in disease.

Although the acquisition of functional input mapping data may be increasingly accessible, analyzing such data is not as straightforward. One of the hurdles faced when analyzing functional imaging data is the need for robust user-invariant routines for region-of-interest (ROI) recognition^35^ and activity data extraction. Progress has been made on this front for somatic calcium transients with a number of shared resources.^36^^–^^39^ However, these code bases have been specifically tailored for processing somatic signals and do not easily adapt to finer structures of dendritic shafts and spines. Dendritic spines have structural characteristics distinct from those of the cell bodies. Clean isolation of the spine heads during ROI detection is the key that prevents the spine activity from getting heavily contaminated by dendritic shaft signals, which exhibit their own distinct activity patterns.^23^^,^^40^ Several, well-developed, open-source MATLAB-based software packages exist that facilitate automated morphological identification and structural quantification of dendritic spines,^41^^–^^43^ but none of these were specifically designed for functional analysis of dendritic spines, nor do they offer comprehensive packages that allow for objective ROI identification, activity data extraction, and activity characterization against other simultaneously recorded behavioral parameters.

To this end, we have developed a custom MATLAB software package for an automated tuning analysis tool dubbed AUTOTUNE, with a user-friendly graphical user interface (GUI), specifically designed for analyzing dendritic spine and local dendritic shaft activity and corresponding stimulus/behavioral feature selectivity. The program not only offers options for both fully automated and point-and-click semiautomated ROI detection modes, but it can also apply the same set of detected ROI seeds on multiple temporal stacks (t-stacks) of images of the same dendrites taken under different conditions or at different times. Moreover, neuronal dendrites have multiple functionally distinct compartments (long dendritic shafts, spines, local dendritic shaft subregions, and branch points) each of which can exhibit different activity profiles and roles in dendritic computation.^44^^–^^46^ AUTOTUNE can independently extract specific transients from all of these compartments. By registering the extracted transients with the stimulus feature time vectors, the software allows for graphing and quantification of stimulus tuning curves and/or correlation plots for each and every detected spine and other functional compartments. To note, while the goal of this study is to provide a fully functioning software that can immediately serve nonprogramming users right out of the package, our software is amenable to customization by proficient coders. This all-around comprehensive software suite, now freely accessible from GitHub (https://github.com/yuyiyi/AUTOTUNE_GUIdevelopment.git), can serve as a primary tool for performing detailed analyses.

## 2 Methods

### 2.1 User and Systems Requirements

MATLAB R2019a or newer is recommended. In addition, the following MATLAB toolboxes are necessary to operate AUTOTUNE: Image Processing Toolbox, Signal Processing Toolbox, Curve Fitting Toolbox, Statistics and Machine Learning Toolbox, and Parallel Computing Toolbox. In order to achieve smooth data processing, operating systems equipped with sufficient random access memory (RAM) and a multicore central processing unit (CPU) are recommended. The software was developed and tested using 32 GB RAM and an Intel Core i7-5960X CPU with a clock speed of 3.0 GHz and an Nvidia GeForce GTX 960 graphics card. Graphics processing unit (GPU) devices is strongly recommended though not required. Under these conditions, we observed that an image stack registration for motion correction could be performed at 93  GB/h. Typical temporal image stacks (t-stacks) we acquire in our lab range from 12,000 frames (512×256  pixels) to 42,000 frames (512×128  pixels) and are 5 to 7 GB in size. These data are motion-corrected in 5 to 15 min on a wide range of machines, including standard laptops.

### 2.2 Software Download and Loading AUTOTUNE

**Fig. 1:**
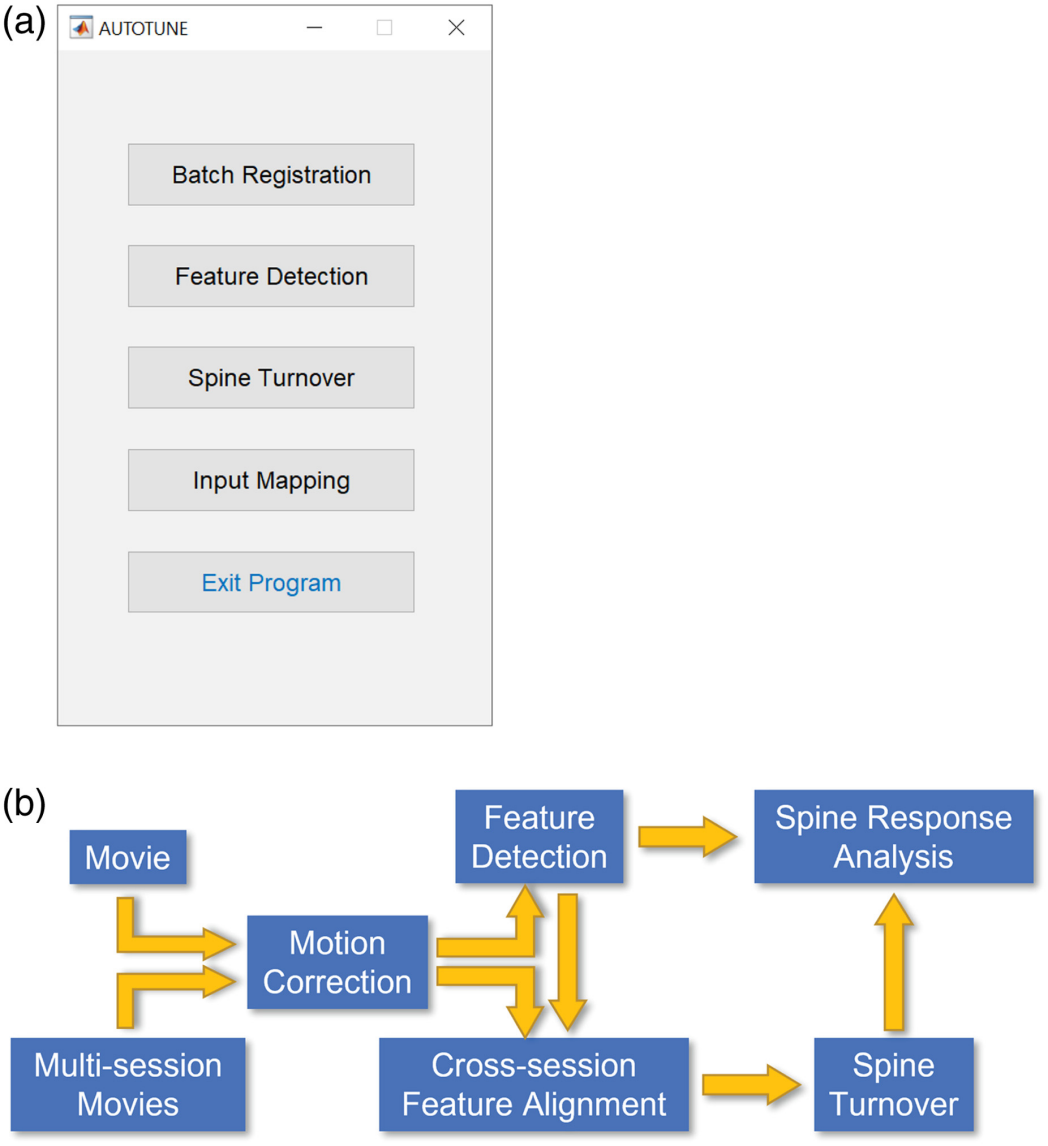
AUTOTUNE features and workflow: (a) AUTOTUNE master control GUI and (b) Analysis workflow of the software. AUTOTUNE offers multiple steps of analyses, many of which can be executed *a la carte*. Functions include motion correction of one or more t-stacks of source images, automatic and semiautomatic segmentation of ROIs, detection of dendritic spine turnover through cross-session feature alignment, and dendritic response tuning analyses.

AUTOTUNE is readily available for download from GitHub (https://github.com/yuyiyi/AUTOTUNE_GUIdevelopment) along with demo sample data files and a user manual (Supplementary Material 2). The master control GUI contains four functional modules: batch registration, feature detection, spine turnover, and input mapping (Fig. 1), each of which will execute a corresponding toolbox with its own prompted GUI. Detailed discussion of these modules is provided in the main text.

### 2.3 Demo Dataset: *t*-Stacks and Stimulus Parameter Vectors

In the GitHub repository, three example image t-stacks (stacks 1, 2, and 3) and three ROI dataset m.files (timepoint 1, timepoint 2, and locomotive behavior) are provided for demonstration purposes. Stacks 1 to 3 were acquired from head-fixed mice using two-photon calcium imaging and contain visually evoked dendritic activity transients spanning 13,500 and 26,000 frames of 16-bit and 8-bit tag image file format (TIFF) images, respectively. For this, layer 2/3 primary visual cortical pyramidal neurons were sparsely labeled with GCaMP7b or 8m^4^^,^^8^ and showed visibly discernible spines. Stack 1 is accompanied by its time course vectors (framestamp) and corresponding visual stimulus parameter matrix (stampinfo; framestamp1). This data pair is provided for the demonstration of AUTOTUNE’s registration, feature detection, and input mapping/tuning response modules. MATLAB m.files, timepoint 1 and timepoint 2, contain ROI feature maps and associated calcium transient data for each ROIs acquired from the same dendrites imaged one week apart. These feature maps were generated from stacks 2 and 3 (also provided so that users can generate their own practice feature maps if desired) and are provided for the demonstration of spine turnover module. Finally, locomotive behavior m.file contains a locomotion-associated dendritic activity dataset, including ROI feature maps and the extracted transients for each ROI, as well as the relevant framestamp and stampinfo appended in it. The stampinfo variables include instantaneous velocity, the virtual linear position of the animal along the treadmill, time courses for port licks and reward dispense, and trial types (open or closed-loop). Locomotive behavior m.file is provided for the demonstration of input mapping/behavioral response module.

It is important to note the key structure in which the framestamp and the stampinfo must be expressed. Framestamp describes the time course vector of the imaging data (i.e., what each image frame is in whatever temporal unit is used in the stampinfo). As such, it should be the same length as the number of frames in the stack or the number of timepoints in the activity transient trace. For example, it can list the exact timing of each image frame in seconds if the stimuli/behavioral parameters in the stampinfo are also expressed in seconds (as used in our demo set for locomotion). Alternatively, it can be integers that indicate the frame number of the image stack (as in our visual response tuning demo set).

Stampinfo accompanying each imaging data contains the time course of a set of stimulus/behavioral parameters. In our visual response tuning demo, the stampinfo contains the time course of the visual stimuli in imaging frames as temporal units. In this stampinfo, eight different grating drift directions are simply expressed as numbers 1 through 8 with 0 indicating gray screen intervals but can alternatively be any real values or categorical strings. In our locomotion demo, the stampinfo contains the locomotion information (instantaneous velocity, the position on the virtual linear track, time courses of port licks, and reward dispense) as well as the behavioral trial conditions (open-loop versus closed-loop) stamped in seconds. The advantage of such structure is that the parameters described in the stampinfo can now have a different temporal resolution or sampling rate, adding degrees of freedom to the experimental design.

### 2.4 Generation of Demo Dataset

#### 2.4.1 Animals and husbandry

All procedures involving living animals were performed in accordance with the guidelines and regulations of the U.S. Department of Health and Human Services and approved by the Institutional Animal Care and Use Committee at the University of California, Santa Barbara. Adult C57Bl/6 mice of both sexes (>8 weeks; Jackson Labs) were housed under a 12/12 h dark-light reversed cycle with *ad libitum* access to food and water.

#### 2.4.2 Surgery

A 4-mm diameter craniotomy was performed over the visual cortex as previously described.^47^ Briefly, mice were premedicated with a sedative, acepromazine (2  mg/kg body weight, i.p.), after which they were deeply anesthetized using isoflurane (2% to 3% for induction and 1% to 1.5% for surgery). The mouse’s body temperature was monitored and actively maintained using an electronic heat pad regulated via a rectal probe. Carprofen (25  mg/kg body weight, s.c.) was administered preoperatively, and lidocaine solution containing epinephrine (5  mg/kg body weight s.c.) was injected locally before and after the scalp excision. The scalp overlaying the right visual cortex was removed, and a custom head-fixing imaging chamber with a 5-mm diameter opening was mounted to the skull with cyanoacrylate-based glue (Oasis Medical) and dental acrylic (Lang Dental) or dental cement (C&B Metabond, Parkell). Mice were mounted on a custom holder via the headplate chamber, which was filled with a physiological saline containing (in mM) 150 NaCl, 2.5 KCl, 10 HEPES, 2 ${\mathrm{CaCl}}_{2}$, and 1 ${\mathrm{MgCl}}_{2}$. A craniotomy was performed using carbide and diamond dental burs on a contra-angle handpiece (NSK).

#### 2.4.3 Intrinsic signal optical imaging and retinotopic maps

In order to functionally map the visual cortex for targeted injection of viral vectors, intrinsic signal optical imaging (ISOI) was performed using a custom microscope and a CCD camera as previously described.^47^^,^^48^ During ISOI, mice were lightly anesthetized with 0.5% isoflurane augmented by acepromazine (2  mg/kg body weight, i.p.), and the body temperature was kept at 37°C. A single-drifting white bar on a black background (3 deg thick, moving in elevation or azimuth direction) was used as previously described^47^ to obtain retinotopic maps based on the intrinsic signals. Retinotopic maps were used to locate V1. The pial vasculature map relative to the retinotopic maps was used to guide targeted injections into V1 at one to two sites.

#### 2.4.4 Viral injections

After craniotomy and ISOI was performed to map V1, adeno-associated viral (AAV) vectors were injected into V1 under continued isoflurane anesthesia as previously described.^19^^,^^23^^,^^47^ Briefly, 1:1 mixture of pENN.AAV.CamKII 0.4.Cre.SV40 [AAV1; Addgene #105558; diluted at 1:20,000 in phosphate-buffered saline (PBS)] and pGP.AAV.syn.FLEX.jGCaMP7b.WPRE (AAV1; Addgene #104493; original concentration at $∼10^{13}\;\mathrm{vg}/\mathrm{mL}$) or pGP.AAV.syn.FLEX. jGCaMP8m.WPRE (AAV1; Addgene #162378; original concentration at $∼10^{13}\;\mathrm{vg}/\mathrm{mL}$) viral particles were injected (65 nL per site; 1 to 2 sites per animal) into V1 with a pulled-glass capillary micropipette using a Nanoliter 2010 controlled by a microprocessor, Micro4 (World Precision Instruments) at 15 nL per min. The glass pipette was left in place for 5 min before retracting to avoid the backflushing of the injected solution. The cranial window was then sealed with a glass cranial plug made up of 4-mm and 3 or 3.5-mm circular coverslips (Warner Instruments) stacked in tandem with a UV-curing optical adhesive (NOA61, Norland).

#### 2.4.5 Visual stimuli for assessing orientation tuning

During two-photon calcium imaging of dendritic orientation tuning responses, the visual stimulus LCD monitor was covered with a cone-shaped shroud with a small opening at the tip that was positioned around the eye of the mouse. This prevented the imaging pathways from getting contaminated with the visual stimulus light. The stimulus extended from +20  deg to +124  deg in azimuth and from −10  deg to +42  deg in elevation. Stimulus frames consisted of 128×128  pixels, which were smoothly interpolated such that one pixel was equivalent to $0.72\;\deg^{2}$ in visual space. Visual stimuli were generated using MATLAB and the Psychophysics toolbox.^49^ Orientation tuning was assessed using drifting black and white square wave gratings as previously described (8 or 12 different directions at 0.04  cycles/deg and 2 Hz.^47^ Each grating was presented for 2 or 3 s separated by 3 or 1 s of gray screen in between, respectively. Each sweep of gratings was repeated 5 to 10 times.

#### 2.4.6 Locomotion

The mouse was allowed to explore a 180-cm long virtual linear maze while headfixed under the objective for two-photon imaging. An encoder was used to feed the locomotion of the mouse to update the instant position within the maze. During closed-loop, the locomotion and the translation of the maze were instantly engaged, while during open-loop, the locomotion and the translation of the maze were decorrelated (the locomotion of a previous trial led the translation of the maze). The virtual environment was displayed in front of the mouse using LED monitors. Three LED monitors were concatenated horizontally by 120-deg angles, which covered the mouse’s visual field that was 150 deg in azimuth and 32 deg in elevation. The position in the maze and the instant locomotion speed of the mouse were recorded. Dendritic spine activity relative to an increase in speed was analyzed.

#### 2.4.7 Two-photon microscopy imaging

Two-photon imaging of ${\mathrm{Ca}}^{2+}$ transients indicated by GCaMP7b or 8m was performed starting 4 to 6 weeks after AAV injection, using a custom-built two-photon microscope used in prior studies.^17^^,^^40^^,^^47^ Our routine imaging sessions were performed using a 16× Nikon objective (N16XLWD-PF, 0.80 NA) with the two-photon laser set to 910 nm at 60 to 100 mW power measured at the objective. Frame scans were acquired using ScanImage^50^ at either 30 frames per second (512×512  pixels), 58 frames per second (512×256  pixels), or 110 frames per second (512×128  pixels).

## 3 Results

### 3.1 Analysis Pipeline Overview

AUTOTUNE is a stand-alone analysis tool that does not require users to have programming expertise. Its GUI has an intuitive design supported with data visualization, progress bars, and timekeeping prompts, which provide real-time updates on the analysis status. The software offers a complete analysis package with multiple steps many of which can be executed *a la carte* according to users’ needs. A typical analysis pipeline would begin with registration of image frames for motion correction, detection of dendritic features, or ROIs [spines, long dendritic shaft (“long dendrite”), local dendritic shaft subregions (shaft subregions)], optional manual proofing of ROIs (both deleting of unwanted ROIs and adding of missed ROIs). Once an ROI map has been generated, it can be applied to datasets from multiple imaging sessions across time and/or treatment, allowing the users to repeatedly identify the same target ROIs. This function is especially useful for longitudinal studies following structural and/or functional changes of the dendritic spines or shafts (see spine turnover). The next procedures are signal extraction from ROIs, and finally, a correlation analysis of extracted signals to temporally corresponding stimulus/behavioral features (e.g., visual or auditory stimulus features, locomotive features, and place fields). Entering “AUTOTUNE” (case sensitive) in the MATLAB Command Window opens the master control GUI with four functional modules: batch registration, feature detection, spine turnover, and input mapping (Fig. 1). Below, we will discuss in detail each of these modules.

### 3.2 Data Format

AUTOTUNE is a flexible application which provides users with an option to either follow the “typical” workflow (Fig. 1) or use the individual modules *a la carte*, as long as the input data meet the requirements of individual modules.

The batch registration module takes in a temporal stack or a series of TIFF files. Images in 8- or 16-bit integers are compatible with the program. The output of the registration module is a MAT-file object, which stores motion-correction parameters and a motion-corrected movie, in either MATLAB binary or TIFF format. MATLAB binary format has the advantage over TIFF in terms of processing speed of read/write routines. If a user wishes, the motion-corrected movie saved in the MATLAB binary files can also be visually inspected using a popular image visualization tool, FIJI (Import-Raw).^51^ The following specific parameters required to open these files on FIJI can be found in the RegParameter file: image length, raw precision, and image size [width, height]. AUTOTUNE also provides users with an option to save a subsampled TIFF file for a quick visual inspection of the motion-corrected output.

The feature detection module takes in either a stack of TIFFs, a sequence of TIFF files, or the MAT-file output from the registration module. Users can either perform feature detection on a raw untreated image file (TIFF) or a motion-corrected movie. The output of the feature detection module is a MAT-file object that contains information about feature locations and the signal time series for each feature. For a “typical” dendritic imaging session, the saved features will include spines, long dendrites and shaft subregions, and their corresponding size, location, time series, and dendritic association for spines and subregions.

The spine turnover module takes in two or more MAT-files that contain dendritic feature maps. Thus cross-session spine turnover analysis relies on the feature maps created through the feature detection module. The output of spine turnover analysis would be saved as a new MAT-file with a default prefix of SpineEvolveAnalysis_.

Finally, the input mapping module loads MAT-files that contain the extracted signal time series of the ROIs. Users can either analyze the features generated by the feature detection module or an existing set of time series in a column matrix format. The output from the input mapping module would be appended to the same input MAT-file. For functional correlative analyses of the extracted signals, temporally corresponding stimulus/behavioral parameters (stampinfo) must be expressed as a table whose first column contains timekeeping information, and then time stamps for the stimulus/behavioral parameters in the rest of the columns. The temporal information for the detected ROI features and stimulus/behavioral parameters should be expressed in the same units as the first column of the stampinfo (e.g., seconds or frames), but the built-in flexibility of the software accepts a different sampling resolution (e.g., two-photon time series at 30  frames/s while the stimulus parameter are at 60 Hz).

All four modules provide an option to perform batch processing, allowing the users to load multiple datasets for a streamlined analysis.

### 3.3 Image Stack Frame Registration for Motion Correction

**Fig. 2:**
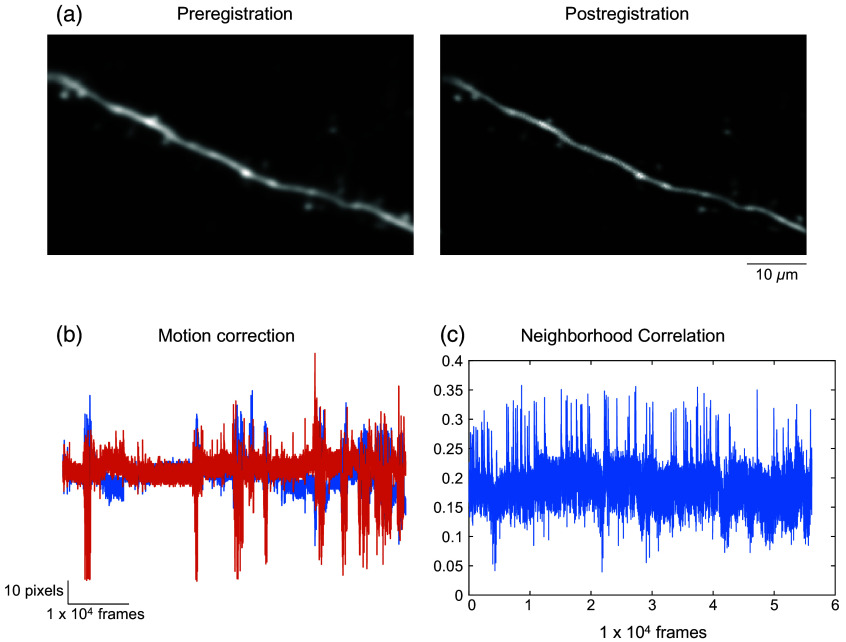
Motion correction with batch registration module. (a) Example frame averaged images pre- and postregistration for comparison. AUTOTUNE generates before and after images for immediate visual inspection. (b) Degrees of frame-by-frame shifts applied during registration. Vertical and horizontal translations in pixels are graphed in blue and orange, respectively. (c) Frame-by-frame correlation coefficient of the neighborhood pixel values is shown. The spiking activity of the imaged dendrite is reflected in the coefficient value as the large correlated increase in the local fluorescence is detected. Larger motion artifacts will generate larger shifts in the frame as can be seen in this example performed on a t-stack imaged from a locomoting mouse.

Shifts in the imaging frames are often encountered during *in vivo* imaging due to biologically induced motions. These shifts can be as rhythmic and subtle as brain pulsation and breathing, or more sudden and pronounced during dynamic animal movement (grooming, locomotion, etc.). Batch registration is an automated toolbox that allows users to correct for these motion-induced shifts in the imaging frames in a batch of files (Figs. 1 and 2). This process can be applied to both multiple unrelated image datasets to be registered independent of one another or to multiple t-stacks of the same field of view (FOV) to be registered and concatenated together into a single ensemble (e.g., concatenating multiple sets of 1000-frame stacks from a single-imaging session but saved into multiple chunks, a conventional method often used to flexibly adapt to the computational power of an end user. It is recommended to save long-imaging session into multistack dataset, as most of the tiff readers cannot open TIFFs larger than 4 GB).

The registration module is adapted from a published toolbox (Suite2p^39^), which employs a phase correlation method in the Fourier domain, which estimates the translation in the X–Y plane. We have adapted the algorithm for images containing sparse fluctuating signals in which image features (e.g., dendritic spines) may only be transiently visible in some frames (Fig. 2). Specifically, we have added two features to accommodate to images with transiently visible signals. First, we have included a searching iteration step during registration initialization, which finds the portion of the stack that is most stable with prominent signals in many of the image features (e.g., during global dendritic signal) and therefore is best suited for initialization. If the average registration correlation is below a set value (<0.2), AUTOTUNE will rerun the initialization with the next stable chunk. Second, motion correction is additionally equipped with safeguard rules for identifying frames that are inadequately registered due to transiently low signals. Inadequate registration of a frame is measured based on set minimum thresholds for correlation value between a registered frame and the target frame, the average signal magnitude, as well as the maximum threshold for xy displacement value. Translation of such a frame would be interpolated from the previous frames. This approach is valid because in a fast time lapse imaging, such as two-photon imaging, the translation between the two continuous frames is low. Default values in the initialization and safeguard settings are defined in and can be changed from util\defaultparameter.m file (see User Guide Section 9 in the Supplementary Material 2).

Registered images can be saved as either compressed binary or TIFF files. Selection of binary files will accelerate the speed of the registration process due to MATLAB’s more efficient native binary read/write routines and is recommended.^52^ The module offers an option to change the maximum binary file size, to better accommodate the computation power available. The maximum allowed file size is automatically predicted, based on the machine. The module also provides the users with an option to save a subsample (% to be set by the user) average-projected registered image for a quick visual inspection. In addition to the motion corrected resultant image, registration performance is also saved in a separate MATLAB file named *RegParameter.mat, in which both frame-by-frame correlations (RegPara. CorrAll) and frame-by-frame x–y displacements are found.

Image registration is the most time-consuming step of the whole process. Use of a GPU can significantly increase the performance speed of the registration and is strongly recommended. Progress percentage (%) as well as the elapsed time in seconds can be tracked in the Command Window of MATLAB.

### 3.4 Robust Feature Detection

#### 3.4.1 Fully automated versus user supervised semiautomated ROI detection

**Fig. 3:**
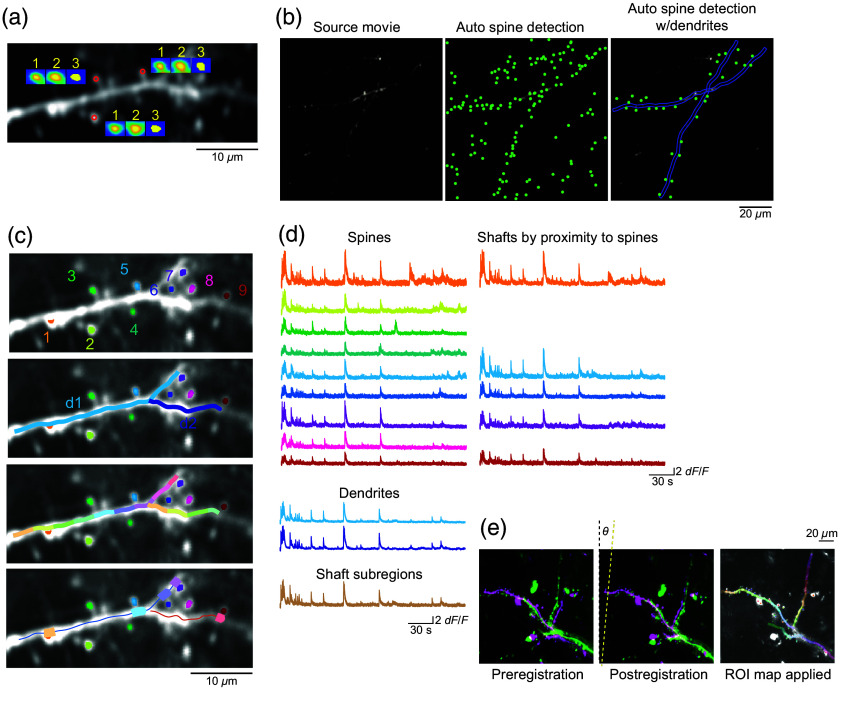
Feature detection and ROI segmentation of dendritic compartments and signal extraction. (a) Detection and segmentation process of three example dendritic spines are shown in insets. The initial ROI (1) detected based on seed pixels defined by the user or automatically defined by a particle detection algorithm is segmented by the signal correlation threshold (2) and then constrained by morphological rules through which features beyond the target size range are excluded. An ROI mask is then generated based on the resultant ellipsoid (3). (b) Autodetection will identify all the ellipsoid puncta available in the image (middle) based first on a particle detection algorithm and then segmented by the spatiotemporal activity profiles of the pixels. Users can also specify a long dendritic branch on which to focus the spine detection. For this latter case, only those spines that are proximal to the target dendrite are detected (right). (c) In addition to spine detection, AUTOTUNE can automatically define local shaft subregion ROIs by the subdivision of the user-defined long dendritic shaft into even pieces or via identification of the shaft subregion located in proximity to a spine ROI. (d) Once ROIs are defined, activity transients can be extracted for each ROI. (e) The same ROI map can be applied to multiple source image stacks of the same FOV via cross-session feature alignment. θ indicates the general angle in which the second stack was rotated to align with the first stack and the master ROIs generated on it.

Accurate measurement of local fluorescent transients of a given biological compartment heavily relies on successful segmentation of ROIs. To this end, we have developed a flexible tool for dendritic spine detection that allows the users to (1) automatically identify all available puncta in the image stack, (2) manually select their preferred ROIs supported by a “point-and-click” style of ROI seed identification, or (3) a combination of the two [Figs. 3(a) and 3(b)]. For all these approaches, AUTOTUNE uses algorithm by which dendritic spine features are segmented based on the correlation map of the intensity transients among neighboring pixels of the seed pixel [Fig. 3(a)]. Users can manually select the seed pixels and/or use the auto spine detection function. The latter identifies seed pixels by a particle detection algorithm based on Gaussian filtering and background intensity estimation.^53^ Neighboring pixels for generating correlation map are defined based on the dendritic width selected by the user, with the segmentation threshold set to 80% quantile of the neighborhood correlation. Autodetection will identify all the ellipsoid puncta available in the image (e.g., dendritic spines and axonal boutons) based on their spatiotemporal activity, unless the user chooses to specify a dendritic branch on which they wish to detect dendritic spines. In the latter scenario, Autodetection will operate under strict criteria, which allows only those spines that are tangentially proximal (default is within 3 times of width of the dendrite set by the user) to the specified dendritic ROIs to be detected [Fig. 3(b)]. In our three representative image t-stacks, autodetection that is unconstrained by the associated dendrite selection had 57.1%±8.0%, 53.5%±1.0%, and 42.9%±8.0% true positives, false positives, and false negatives detected, respectively, compared to the ground truth set by manual ROI selection by a human expert. Autodetection with the associated dendrite selection had 58.3%±6.6%, 4.6%±2.9%, and 41.7%±6.6% true positives, false positives, and false negatives detected, respectively (Fig. S1 in the Supplementary Material 1). The higher incidents of false negatives than false positives in the latter approach are expected as the autodetection is set with stringent parameters (threshold set 80% quantile neighborhood correlation within 3 times of dendritic width in pixels). We set these parameters because it makes for a smoother user experience to manually add missing spines than to delete phantom spines from the images with annotations on the GUI. Because the autodetection of spines with a specific dendrite selected restricts its search area within three times of the dendritic width set by the user, users are encouraged to explore and set the dendritic width specific to their dataset that provides the best results.

Unrestricted autodetection can be applied on a batch of multiple registered image stacks but will produce independent ROI map for each. Alternatively, users can opt to use an existing master ROI map, which allows them to realign multiple stacks and apply the single master ROI map to all of them. In contrast, the manual feature detection option will allow users to manually identify long dendritic branches and dendritic spines as well as delete unwanted features including those puncta that were automatically detected using Auto Detection.

In addition to spine detection, AUTOTUNE offers three different methods in which dendritic shafts may be identified [Figs. 3(c) and 3(d)]: (1) user defined identification of a long dendritic shaft (add dendrites), (2) automated subdivision of identified long dendritic branch (subdivide dendrite), and/or (3) identification of dendritic shaft subregion located in physical proximity to an already detected and accepted spine (shaft by spine). The length of each subregion is set by the user in pop-up window. Local dendritic shaft subdivision is especially useful for probing for local dendritic spike-associated changes that are distinct from signals associated with the backpropagating action potentials.^23^^,^^40^

Once accepted, all the ROIs (long dendritic shaft, local shaft subregions, and spines) are displayed on ROI result box with extracted traces stack plotted in the respective boxes for dendrites and spines [Figs. 3(c) and 3(d)]. After inspecting the feature map, any unwanted features, both dendritic shaft and spines, can be deleted with delete feature function. Once an ROI map is generated, users can use it for subsequently acquired data from the same FOV.

#### 3.4.2 Cross-session feature alignment

One of the great advantages of fluorescent imaging is the ability to monitor both the dynamics of dendritic and spine morphology and functional activity across multiple imaging sessions. These dynamics include those introduced through natural physiological changes, such as learning and aging as well as others associated with the progression of neurological disorders. Although prior studies have mainly focused on morphological changes, accelerated improvements in GECIs and other indicators have enabled chronic functional monitoring of these subcellular compartments. AUTOTUNE’s feature detection tool allows for batch application of the same master ROI map to image sets acquired over multiple sessions. The user will first use the module, feature detection, on one of the registered image t-stacks to generate a master ROI map (e.g., dendritic branches, spines, and local dendritic shafts), which will be then applied to all the registered stacks simultaneously using the batch feature detection module.

**Fig. 4:**
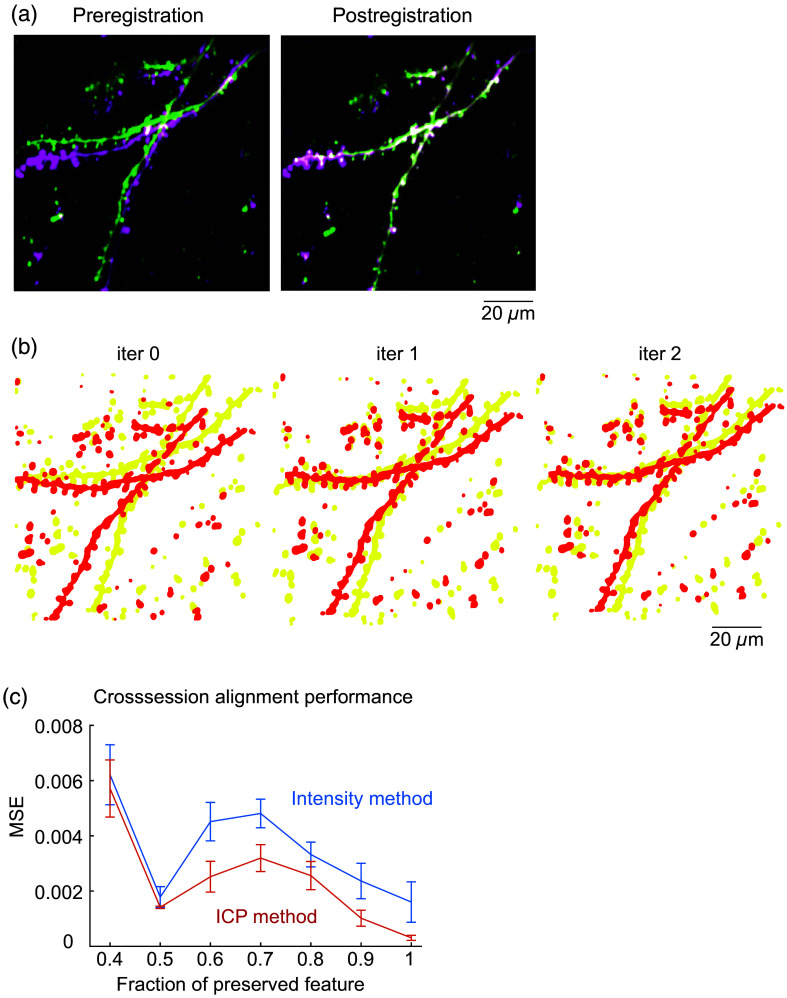
Cross-session image alignment. (a) Example pre- and postregistration overlays of frame averaged images from two stacks of source images acquired during two separate imaging sessions 1 week apart. (b) Automatically detected key feature maps are first defined for each stack (iter 0), which then are shifted closer together at each iteration (iter 1, 2, …), allowing the program to progressively, but simultaneously correct for displacement and rotation. (c) A simulative quantification showed that ICPs correction used by AUTOTUNE demonstrated superior performance generating significantly less error (MSE, mean square error) compared to a built-in MATLAB function for intensity-based automatic image registration (imregtform). In this test simulation, arbitrary translation and rotation were applied to an image in order to quantify the performance of two registration methods. The fraction of preserved features is a portion of image features that remained within the simulation window after the forced translation and rotation.

Functional images from the same FOV acquired across multiple sessions, days, weeks, or even months apart, can contain larger shifts in ROI locations due to physiological changes over time. For such cross-session comparisons, AUTOTUNE employs the iterative closest point (ICP) algorithm for rigid point cloud-based registration in order to apply a single master ROI map onto each stack.^54^ This process allows users to keep track of the same ROIs in longitudinal studies rather than having to cross-reference multiple maps. Theoretically, the amount of pixel shift in X and Y axes is unlimited [Fig. 3(e)]. However, a realistic limitation applies because features can be lost out of frame with large shifts (Fig. 2). In order to test the versatility of our method, we compared the cross-section alignment performance of AUTOTUNE to the MATLAB built-in function for intensity-based automatic image registration (imregtform^55^). Our batch registration method significantly outperformed its counterpart (p=0.001, t-test; Fig. 4).

### 3.5 Longitudinal Study of Spine Turnover

**Fig. 5:**
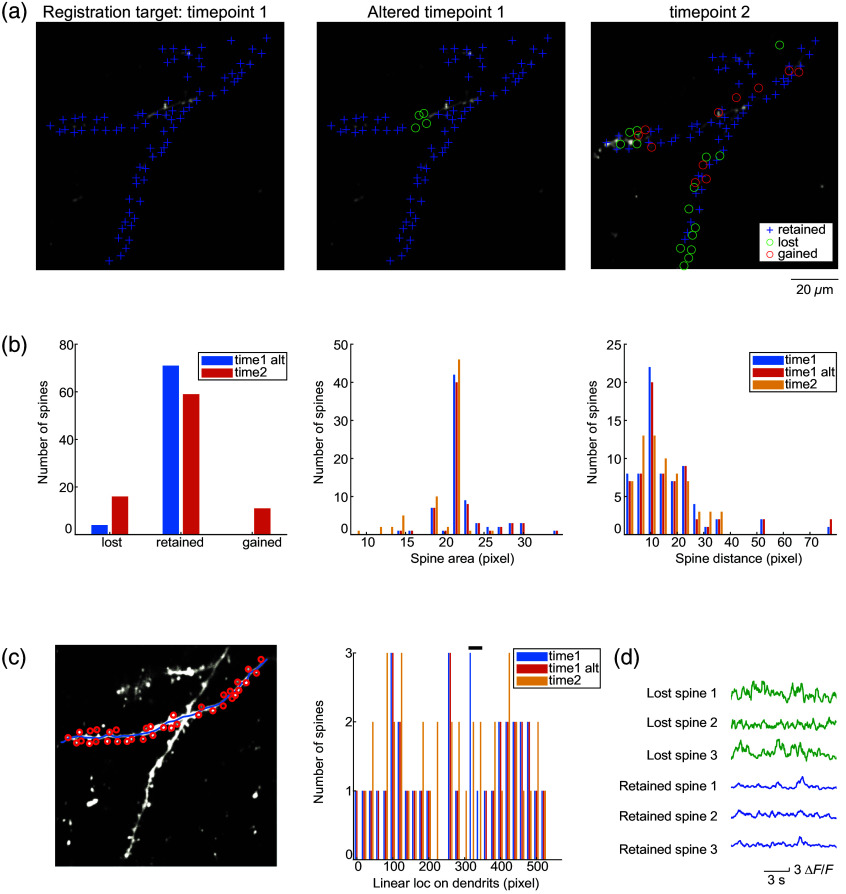
Cross-session spine turnover analysis. (a). Example spine turnover results are shown, comparing the ground truth dendritic spine ROI map manually identified by a human expert from the image acquisition session 1 (left: timepoint 1) to another map from the same session from which four spines were artificially removed (middle: altered timepoint 1) and a map from session 2 taken a week later from timepoint 1 (right: timepoint 2). AUTOTUNE automatically categorizes the spines into three groups via cross-session feature alignment: lost (O), retained (+), and gained (O). (b). Numbers of lost, retained, and gained spines from the example image comparisons in (a) are shown alongside two histogram showing spine ROI area and nearest-neighbor distance distributions. These graphs are automatically generated by AUTOTUNE. (c) Comparisons of linear locations of spines along the associated dendrite are also automatically graphed. Black bar at the top indicates the location from which four spines were artificially removed from the altered timepoint 1. (d) Activity transients from any of the defined ROIs are readily extracted. Extracted transients from three lost spines and three retained spines are shown.

Being able to perform cross-session feature alignment means that users can now perform spine turnover analyses with AUTOTUNE. For this module, the user will first generate ROI maps for each of the functional t-stacks acquired on different sessions or timepoints. For objective handling of the data, we recommend that the users be blind to the specific timepoints of the images. Performing cross-session feature alignment on independent ROI maps for respective image stacks will allow the users to then automatically survey and categorize the spines into three different groups: lost, retained, and gained [Figs. 5(a) and 5(b)]. To correct for potential positional shifts of the dendritic features over time, AUTOTUNE allows the users to select their own constraint value in pixels that defines a displacement distance beyond which two ROIs compared cross session are considered independent. For example, if one sets this value to be 4 pixels, two spine ROIs from different sessions are considered the same and therefore categorized as “retained” if the shift in their centroid location is ≤4 pixels. If it is >4 pixels, then they are considered two different spines and therefore the original spine is marked “lost.” A new spine is registered “gained” even if its location is within the set displacement value when preexisting spines in the vicinity are all accounted for. An appropriate displacement distance (in pixels) should be applied based on the image resolution as well as spine size and density (Fig. S2 in Supplementary Material 1). The module will automatically generate a bar graph containing the total counts of spines categorized as retained, lost, or gained [Fig. 5(b)]. In addition, three histograms showing the spine ROI area distributions, nearest-neighbor spine distance (local spine density), and spine spatial distributions along the dendrite are displayed [Figs. 5(b) and 5(c)]. These graphs are designed to help visualize structural and/or spatial distribution patterns that may be present in the spine turnover.

Most importantly, AUTOTUNE compiles the spine turnover results in a data file called “spine_evolve,” which has all the individual spine ID numbers and their turnover status. Users can use these IDs to then find the respective activity data saved during feature detection (see the operational guide in Supplementary Material 2). Further inspection of such activity profile of the specific spine ROIs that turned over will provide insights into the functional properties of those spines that met the specific fate compared to others [Fig. 5(d)].

### 3.6 Functional Characterization of Synaptic Inputs and Local Dendrites

#### 3.6.1 Removal of back-propagating axonal action potential signals

**Fig. 6:**
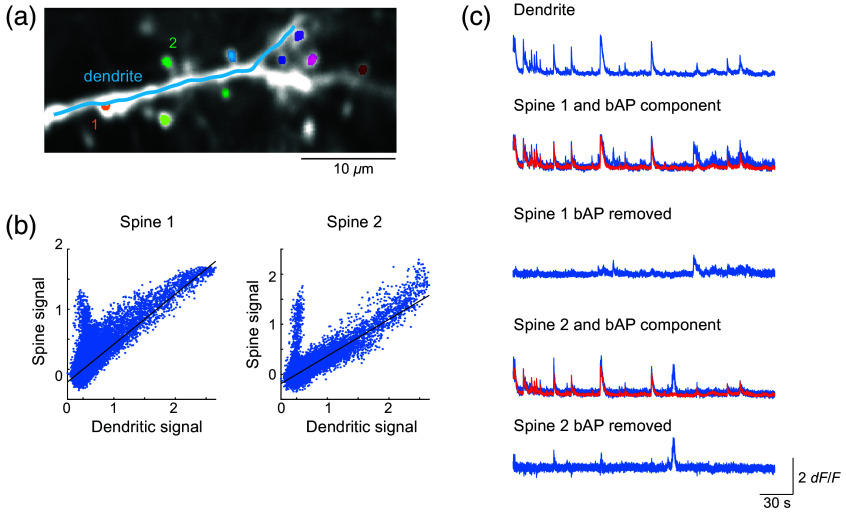
Removal of bAP for isolating input-specific spine signals. (a) ROI map consisting of a single long dendrite and nine dendritic spines are shown. (b) AUTOTUNE applies robust fit function to the correlated portion of the spine and dendritic activity in order to estimate the scale factor (slope) for the back-propagating action potential (bAP) signal contributing to the spine signals. (c) The dendritic activity trace was scaled down by the factor calculated in (b) (red trace) and subtracted from the two example spines, 1 and 2, to reveal the respective input-specific spine signals.

Once the activity traces of the individual ROIs have been extracted, the fourth and final module will allow the users to correlate the trace to any temporally matched parameters, including stimulation features (e.g., orientation of gratings and local optical features in natural images) and other behavioral parameters (e.g., locomotion features, such as speed or location). For mapping dendritic spine calcium signals, the first step that is critically important is to remove any signal contributions from the back-propagating action potentials (bAPs). For this, we have adopted the method by Chen et al.,^2^ in which a linearly scaled version of a long dendritic shaft signals is subtracted from the spine signals^2^^,^^19^^,^^23^ (Fig. 6). Briefly, visually evoked dendritic responses are plotted against those temporally matched responses from each dendritic spine [Fig. 6(b)]. The resultant scatter plot should exhibit two distinct clouds of points; one extending vertically along y axis around x=0 and the other extending diagonally. The former indicates spine responses that are independent of the dendritic shaft responses, and the latter represents instances in which both spine and the shaft were activated simultaneously, inferring the presence of bAPs. MATLAB’s robustfit function is then applied to the latter group of points, and the slope of the fit is used as the factor for scaling the raw dendritic shaft signals to be subtracted from the spine signals. This process is performed in a batch where appropriate scale factors are set for all individual identified spine ROIs using the accompanying long dendritic shaft signals. Although the process has been well-adopted by other published works that have advanced our knowledge of dendritic spine physiology,^2^^,^^19^^,^^22^^,^^23^^,^^56^ a care must be taken as a simulation has reported potentially biased results created by over- or undersubtraction of bAP contributions.^57^ AUTOTUNE offers immediate visual feedback on the resultant bAP-removed trace, allowing the users to select the largest scale factor (to minimize undersubtraction) for each spine (and local shaft subregion) that does not generate negative deflections (to prevent oversubtraction) [Fig. 6(c)]. Use of the dendritic activity rather than the somatic activity as a bAP activity proxy and exclusion of shaft subregions that exhibit visible nonlinear events (i.e., calcium hotspots from local dendritic spikes) are two important additional considerations that should be taken to minimize the potential biases in the results (Fig. S3 in Supplementary Material 1). bAP removal process can be entirely omitted for signals that do not convey bAPs such as those detected with glutamate sensors.^1^ After contaminating bAP signals are appropriately subtracted from the spines, users can perform correlative analysis of the spine signals with a temporally matched stimulus or behavioral parameters of their choosing.

#### 3.6.2 Input mapping: orientation tuning analysis of dendritic spine responses

**Fig. 7:**
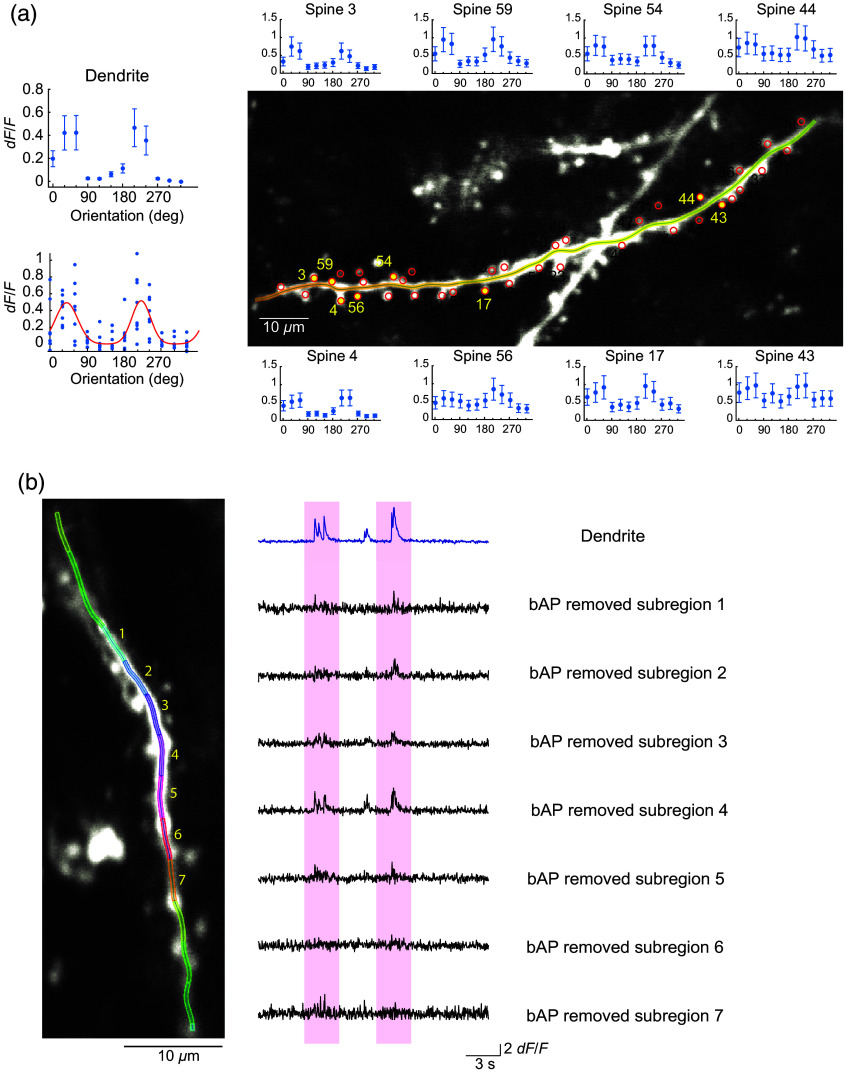
Orientation tuning analysis of dendrite and spine responses. (a) AUTOTUNE was used to analyze the orientation tuning of long dendrites and spines. An example dendrite exhibiting sharp orientation preference is shown. The tuning curve was fit with a constrained two-peak Gaussian tuning curve (red). Individual spines also exhibited visually evoked calcium transients and displayed tuned responses of various strengths. (b) Spatially localized dendritic activities were discernible in bAP-removed traces of shaft subregions, potentially indicating incidents of local dendritic spikes.

**Fig. 8:**
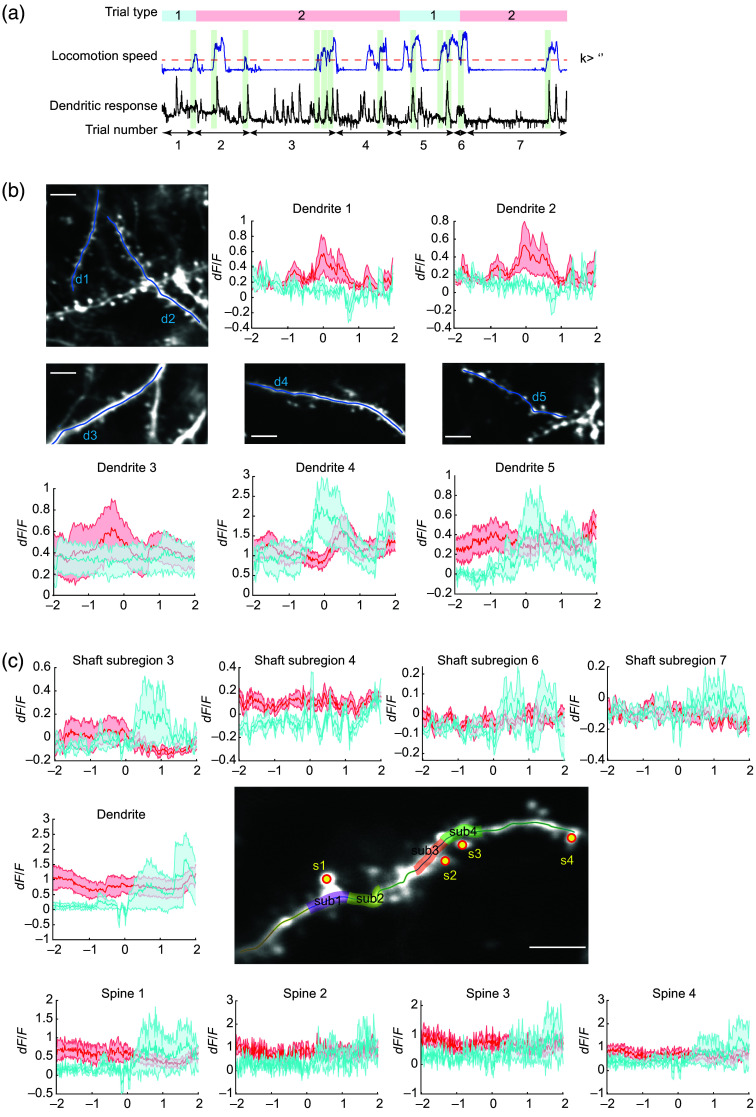
Probing for behavioral correlations in spine activity. (a) Schematic showing the trial structure of an example dendritic imaging experiment during a visually guided locomotion task is shown. As a head-fixed mouse walked along the linear virtual treadmill, locomotion speed (instantaneous velocity: blue trace) and dendritic calcium responses (black trace) were recorded. Each trial was considered complete as the mice traveled to the end of the virtual path. The locomotion of the mouse was instantly translated to its position in a virtual track marked with visual cues (closed-loop: red blocks in trial type) or disengaged from the visual feedback (open-loop: blue blocks). AUTOTUNE input mapping/behavioral response module can plot event-triggered averages of the dendritic activity (green shaded area) using a transition in the locomotion speed (e.g., k > value set by the user: red dotted line) and compare between the two trial types. (b) Event triggered averages of calcium transients recorded from five dendrites as mice (n=2) accelerated its locomotion (k>0.5  cm/s), comparing between the open- (blue) and closed-loop (red) trials are shown. Time 0 s indicates the initial time of acceleration. (c) Locomotion speed triggered averages of calcium transients from a dendrite, four shaft subregions, and four spines are shown. The x axis is in seconds; scale bar=10  μm.

In this study, we offer two example cases in which our program computes correlative relationships between the spine activity and the stimulus or behavioral features, namely, moving direction of oriented black and white gratings (orientation tuning) and locomotive speed, respectively (Figs. 7 and 8).

For mapping visual response properties of synaptic inputs arriving at dendritic spines of mouse V1 layer 2/3 pyramidal neurons, AUTOTUNE was used to analyze their orientation tuning. In general, imaged neurons exhibited strong visually evoked activities reflected as global dendritic shaft spiking activities (Fig. 7). An example dendrite showed sharp orientation preference and could be fit with a constrained two-peak Gaussian tuning curve. Individual spines also exhibited visually evoked calcium transients and displayed noticeable tuned responses [Fig. 7(a)]. Local shaft subregion responses were also observed in another example dendrite with evenly subdivided shaft ROIs [Fig. 7(b)]. In this example, spatially localized shaft subregion activities were discernible in bAP-removed traces, potentially indicating incidents of local dendritic spikes.^23^^,^^40^ These datasets demonstrate the ease and the effectiveness of AUTOTUNE.

#### 3.6.3 Input mapping: application of dendritic analyses to behavioral settings with multiple stimulus variables

The input mapping module allows us to probe for the circuit-based mechanism of synaptic integration by closely examining potential correlative relationships between dendritic spine responses with complex experimental parameters, such as behavioral parameters with or without stereotyped trial structure (e.g., duration and condition). Here we provide an example test case for how this type of analysis may be performed with AUTOTUNE on dendritic imaging acquired while the mouse traveled in a linear virtual treadmill.

AUTOTUNE enables the comparison of spine activity under different conditions of a select parameter. In this test case demonstration, we analyzed the dendritic activity against the locomotive velocity, focusing on the 4 s window centered around the point at which the animal transitioned from stationary to moving (e.g., instantaneous speed >0.5 cm/s). We used AUTOTUNE to generate event-triggered averaged trace of a long dendritic shaft, shaft subregions, and spines spanning 2 s before and after the transition, which allowed us to explore the potential impact of locomotion on dendritic activities. Moreover, AUTOTUNE allows for trial-structure-based categorization of event-triggered averages for a group comparison [Fig. 8(a)]. The example case compared the locomotive speed-triggered averages of dendritic and spine activities between open-loop and closed-loop trials as the mice navigated a virtual treadmill through a simple visual scene [Figs. 8(b) and 8(c)].

The behavior analysis module is versatile and allows users to define the window size of the trace snippet to inspect, according to their rationale and needs. In addition, for more complex experiments with multiple trial conditions, AUTOTUNE allows users to perform joint analysis on how certain parameter affect neuronal responses under different trial conditions (Fig. 8).

## 4 Discussion

The field of systems neuroscience has always been invigorated and advanced by biologists of creative minds. As the field has advanced, the bottleneck has perhaps shifted from the creativity of the experimenters to technological availability and computational handling of large-scale datasets. With the recent advances in commercially available multiphoton microscopes and fluorescent indicators of neuronal activity (e.g., GECIs and GEVIs^2^^–^^5^), functional imaging of neuronal activity, especially for mapping of synaptic inputs, has become increasingly accessible to many and is bound to further our understanding of the neural functions, including linear and nonlinear synaptic integration in individual neurons. The hurdle that remains is the computation-heavy analyses of the acquired data. The goal of this study is to breakdown these barriers by offering an analysis software suite with a user-friendly GUI that can be easily accessible to nonprogrammers.

New types of data often require custom software. Although there are many software suites for analyzing two-photon calcium imaging data from cell bodies, functional synaptic imaging involves a number of unique challenges. To the best of our knowledge, AUTOTUNE is the first open-source software suite to address these challenges. Our software, AUTOTUNE, specifically designed with analyzing *in vivo* dendritic and spine activity in mind, offers semiautomated analysis pipeline that assists the users from image stack preparations with high-precision frame registration (motion correction) through ROI detection, signal extraction, back-propagating axonal action potential subtraction, and response property quantifications. The GUI is designed to be intuitive, with rapid feedback and semiautomated tools to ensure user control and supervision of the analysis. Quality control and spot checks are critical during analysis, and thus we opted for a semiautomated workflow. This can limit throughput, but it is essential for controlling and ensuring that invalid ROIs are excluded from further analysis. Moreover, permitting a supervised semiautomated workflow relaxes the constraints on the algorithms. We can use greedy algorithms to segment many potential spines and then exclude those that do not pass a human examiner. Overall, in practice, this approach can result in higher throughput, with reduced demands on the computing time with simpler algorithms. Blind analysis and cross checking with multiple experts can be additionally used to ensure rigorous inspection of the ROIs.

The software suite can be used in synaptic calcium imaging experiments out-of-the-box by naïve users. Alternatively, advanced programmers may benefit from using the software for conducting a quick survey of the spine signal properties in order to inform their next step in designing further advanced signal analyses that may be more specific to their needs. The code is based in MATLAB, which is widely used in the community for the analysis of functional two-photon imaging data from neuronal cell bodies. A port to Python is feasible, as has been done for some other scientific software suites for calcium imaging analysis (e.g., Suite2p, which started in MATLAB and now has a Python version^39^).

In this study, we presented data, demonstrating the utility and effectiveness of AUTOTUNE. We provided demo analyses of dendritic and spine imaging in layer 2/3 pyramidal neurons in mouse V1, which has been a key proving ground for functional synaptic imaging technology.^19^^,^^20^^,^^22^^,^^23^^,^^56^ While the demonstration was performed using calcium imaging data acquired during visual stimulation, AUTOTUNE could be easily applicable to analyzing fluorescent transients generated by other sensors, such as neurotransmitter sensors^1^ and voltage sensors^3^ in response to other sensory modalities (e.g., somatosensory and auditory inputs). Users are strongly encouraged to be familiar with the temporal kinetics of the indicator of their choosing in order to select an appropriate frame rate for imaging. For example, while GCaMP signals may be resolved at frame rate below 30 Hz, imaging speed at or above 100 Hz is required for glutamate sensors such as iGluSnFR3.^1^ However, once that is achieved, our ROI detection tool is uniquely robust against a wide range of indicator-specific kinetics because unlike some of the other ROI detection tools, the early phase of AUTOTUNE’s feature detection module (i.e., automated ROI seed detection) is based on an averaged image intensity and does not rely on temporal information. In fact, AUTOTUNE can be used for detecting static puncta in a 3D volumetric stack with the z axis being the vertical steps on different planes of focus (puncta labeled with KikGR;^58^ Fig. S4 in Supplementary Material 1). The module will only access the temporal information in the final phase when it computes the neighborhood correlation with the seed pixel. However, because AUTOTUNE was designed and optimized specifically to handle the functional activity of dendrites recorded in temporal time-series movies, it has the following limitations that the users should be aware of: (1) AUTOTUNE cannot be used for registration of single-frame static images, (2) it cannot be used for 3D volumetric stack registration of static structural images, and (3) its algorithms are not equipped to handle 3D volumetric stack of functional data (e.g., images acquired with a Piezo objective scanner).

AUTOTUNE is open-source and extendable for diverse functional synaptic imaging experiments. The freely available code may additionally serve as training tools for those who aspire to learn coding relevant to the systems neuroscience.

## 5 Appendix: AUTOTUNE Operational Manual

The operational guide for AUTOTUNE is included in Supplementary Material 2.

## Supplementary Material





## Data Availability

The archived version of the code described in this article can be freely accessed along with a set of demo data and a step-by-step manual in GitHub at https://github.com/yuyiyi/AUTOTUNE_GUIdevelopment.git.
