# Supplementary material for: Comprehensive software suite for functional analysis and synaptic input mapping of dendritic spines imaged in vivo
Source: Neurophotonics. 2024 Apr 16;11(2):024307. doi: 10.1117/1.NPh.11.2.024307 (PMC11021036; doi:10.1117/1.NPh.11.2.024307)
Supplement: Supplementary file 1 [file NPh_011_024307_SD001.pdf]

Supplementary Figure S1

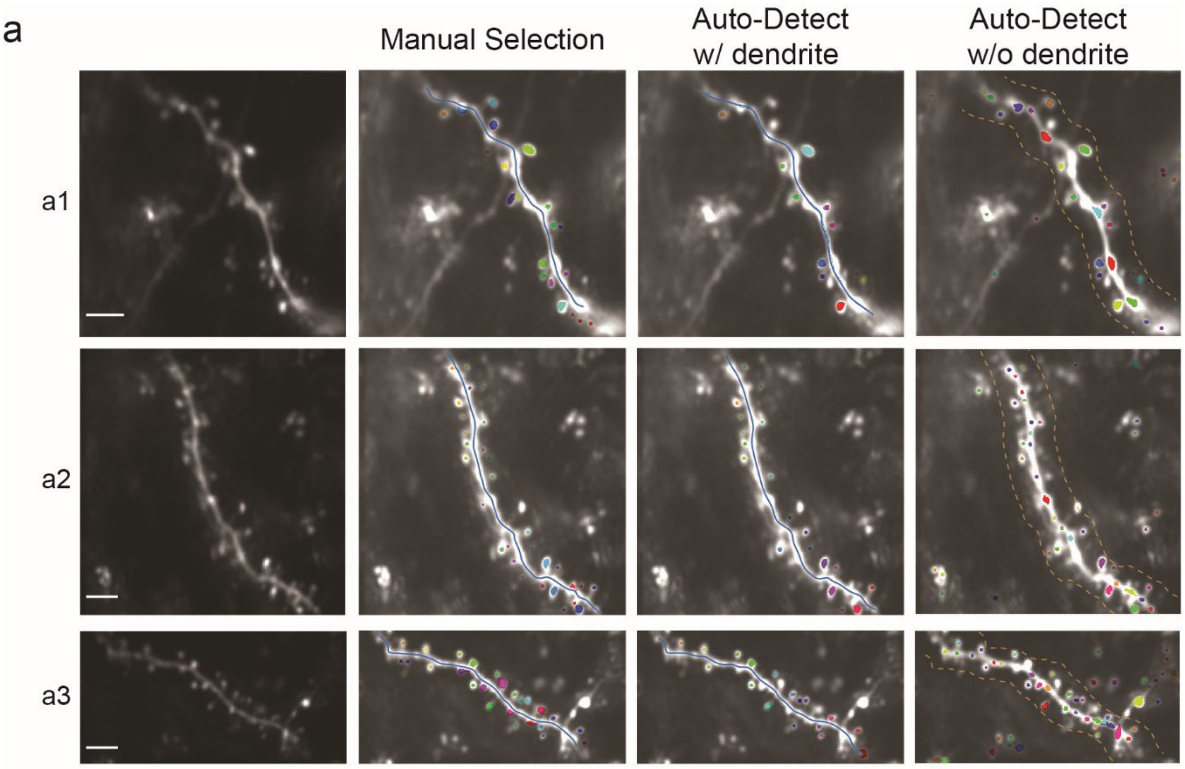

b

Stack ID	Total Spines	Auto-Detect with dendrite specified			Auto-Detect without dendrite		
		True Positive	False Positive	False Negative	True Positive	False Positive	False Negative
a1	20	9	2	11	12	11	8
a2	31	20	0	11	13	16	18
a3	26	17	1	9	18	14	8

Supplementary Figure S1. Detection rates of AUTOTUNE's Auto-Detect functions. a. In three representative image t-stacks (a1 through a3), spine Autodetection results using two different algorithms available in AUTOTUNE were compared to the ground truth provided by a human expert (Manual Selection). Colored ellipsoids and blue lines indicate detected spines and a target dendrite in manual selection and the constrained Auto-Detect mode (w/dendrite) in which a spine search area that is tangentially proximal to a selected dendrite, respectively. Orange dotted lines on the image-wide detection results by the unconstrained Auto-Detect mode (w/o dendrite) indicate the typical nearby area to the target dendrite the expert would constrain their search. The area within these orange-dotted bounds were used for quantifying the performance of the unconstrained Auto-Detect mode. **b.** True positive, false positive, and false negative spines were counted for each condition. Note that many of the false positive spines for the unconstrained Auto-Detect mode were found on top of the dendritic shaft. scale bar = 10μm

Supplementary Figure S2

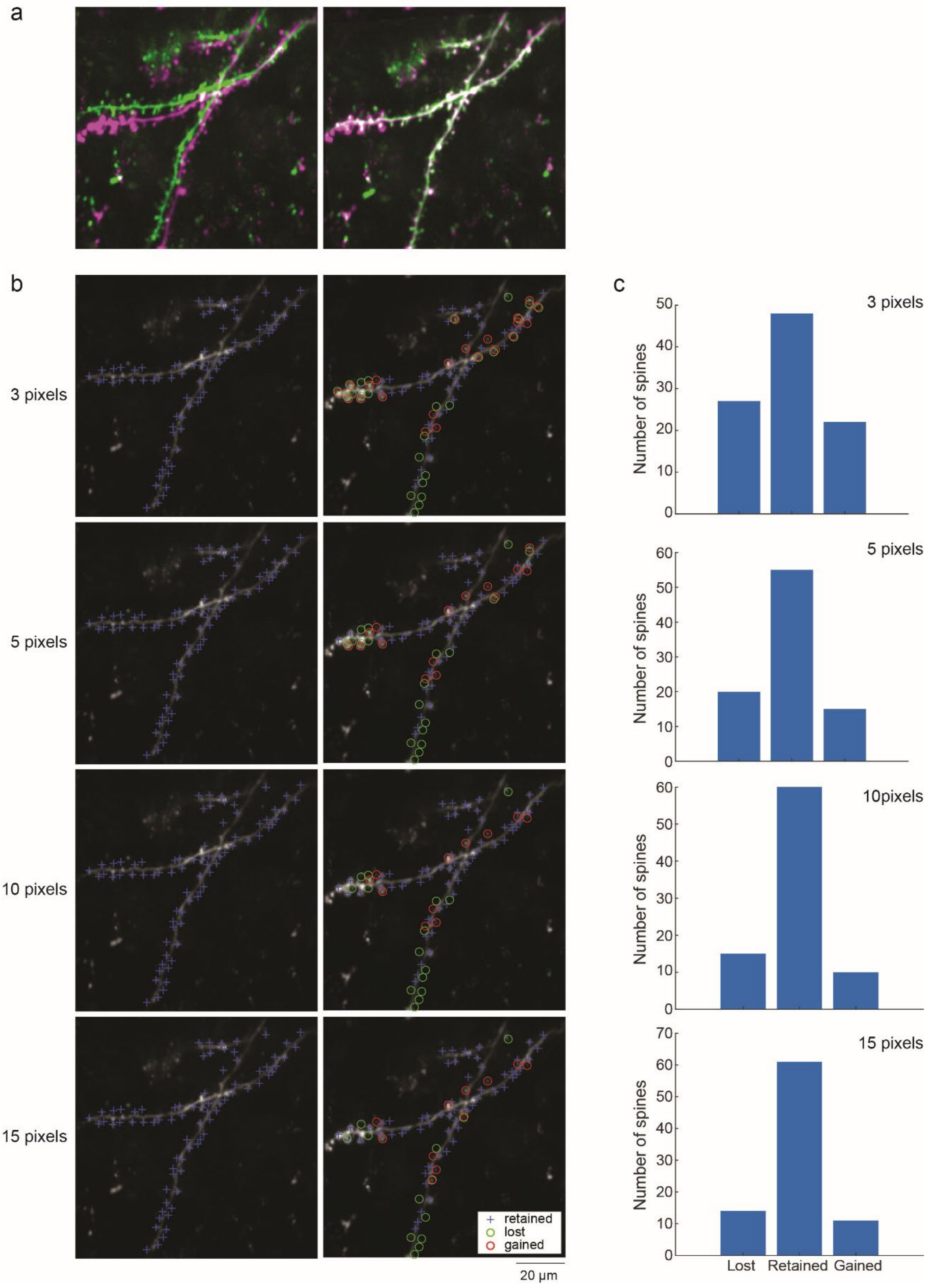

Supplementary Figure S2. Impact of pixel values for defining independent spines. **a.** An example cross-session registration result of two t-stacks (timepoint 1 in green vs. timepoint 2 in magenta) imaged a week apart is shown. **b**. The effects of four different pixel definitions for spine independence are shown. c. Pixel values that are too small (e.g. 3 and 5 pixels) result in miscounting of retained spines as lost or gained where as values that are less stringent (e.g. 15 pixels) may result in overestimation of retained spines. **c.** AUTOTUNE automatically graphs the spine counts for each category.

Supplementary Figure S3

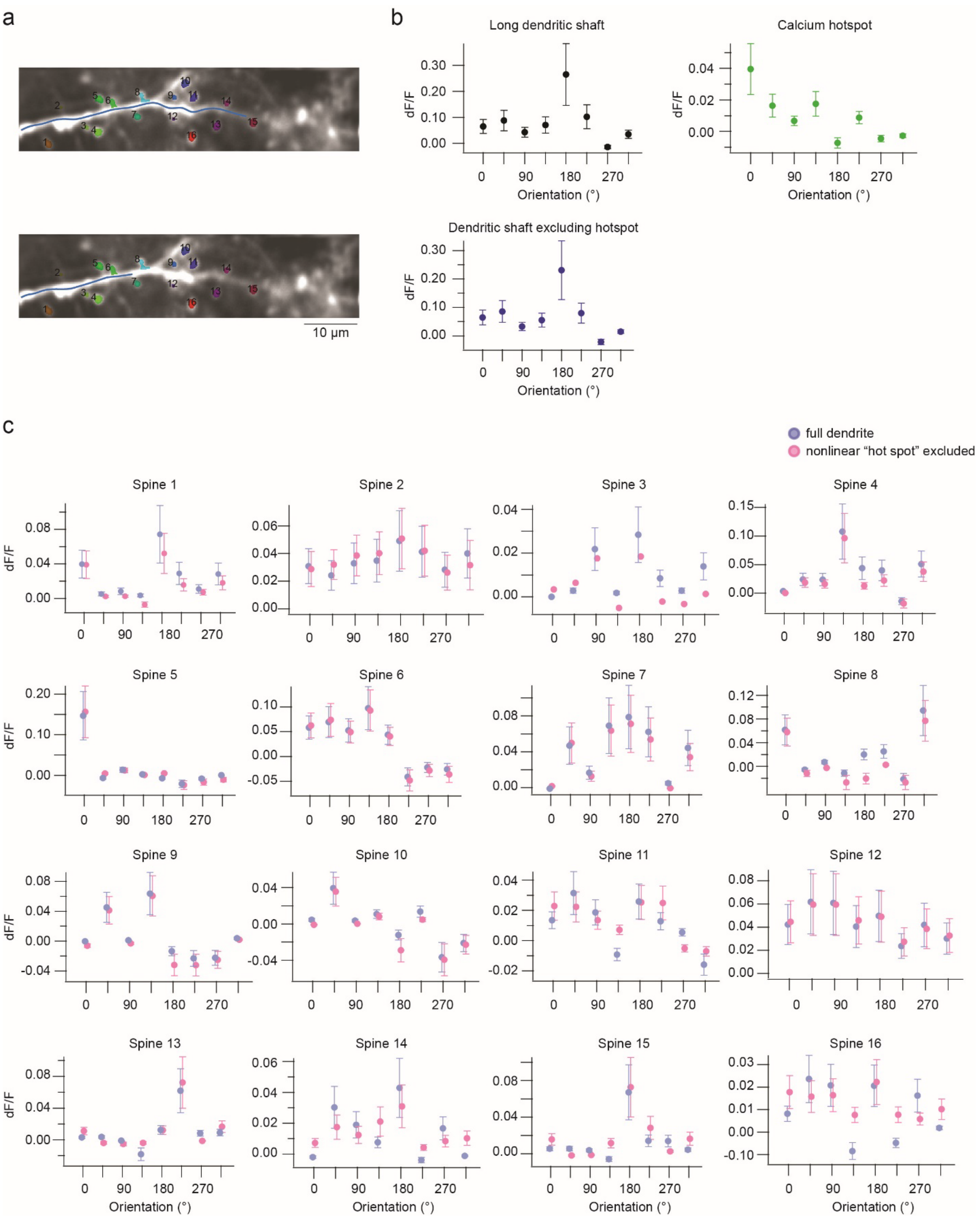

Supplementary Figure S3. Impact of local nonlinear dendritic events (calcium hotspots) on bAP removal process. a. ROI feature maps for an example dendrite that exhibited a stretch of calcium hotspot in the local dendritic shaft subregion are shown. Top: dendritic ROI was drawn across the entire stretch including the subregion that showed the nonlinear event. Bottom: dendritic ROI was drawn excluding the hotspot. AUTOTUNE was used to analyze the orientation tuning of long dendrites and spines. **b.** Orientation tuning plots of the whole dendrite, a shorter dendrite excluding the hotspot, and the hotspot itself are shown. Note that in this case, the two dendritic tuning curves (black and blue) were similar, suggesting that the impact of differently tuned calcium hotspot on the overall dendrite tuning was negligible. **c.** Tuning plots of 16 spines were compared between two scenarios in which signals from the full dendrite visible within the image frame (blue) or those from a shorter dendrite excluding the calcium hotspot (pink) were used for bAP removal process. In most of the spines (14/16 total), the preferred orientation between the two scenarios did not change. Two spines (spine 11 and 16), however, showed some differences in the preferred orientation.

Supplementary Figure S4

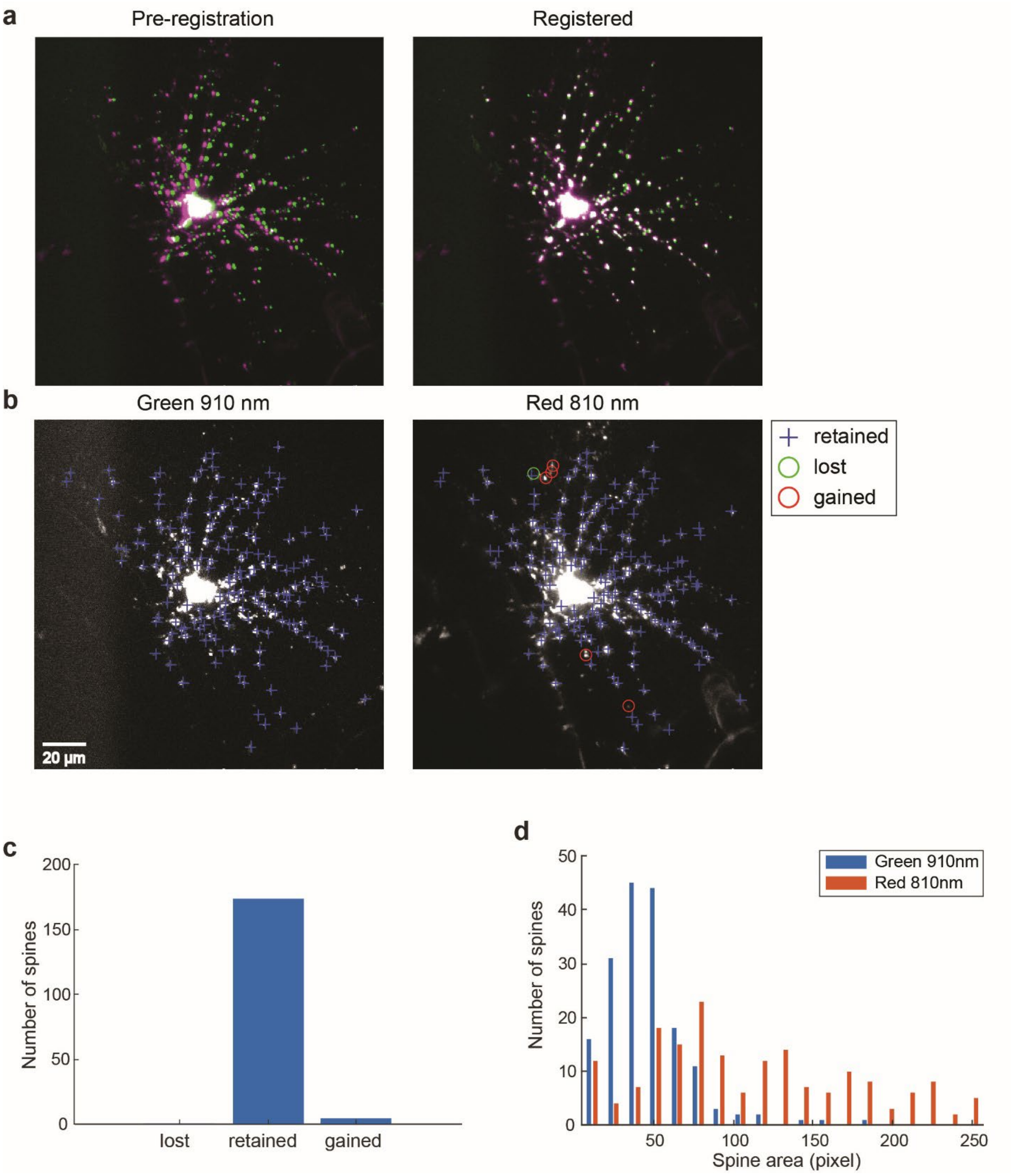

Supplementary Figure S4. Example case in which AUTOTUNE was used for cross-session registration and turnover analysis of fluorescent punctate in a static fluorescent z-stack. a. A cross-session registration result of two z-stacks of the same neuron expressing photoconvertible KikGR in a punctate pattern across dendritic arbor before and after the conversion is shown. **b-d**. In this preparation, kikGR underwent green-to-red conversion after 2min UV exposure. Green fluorescence before the conversion was imaged at 910nm and red fluorescence that appeared after the conversion was imaged at 810nm (**b**). While the punctate that are observed are not confined to dendritic spines, AUTOTUNE's Spine Turnover module was still able to identify most of the KikGR punctate as "retained" (**c**), and the "spine area" measurement function could be applied to quantification of the puncta size distribution (**d**).
