# Supplementary material for: Comprehensive software suite for functional analysis and synaptic input mapping of dendritic spines imaged in vivo
Source: Neurophotonics. 2024 Apr 16;11(2):024307. doi: 10.1117/1.NPh.11.2.024307 (PMC11021036; doi:10.1117/1.NPh.11.2.024307)
Supplement: Supplementary file 2 [file NPh_011_024307_SD002.pdf]

DENDRITE

USER GUIDE FOR AUTOTUNE

Step-by-step manual on how to use AUTOTUNE modules

FEBRUARY 20, 2024

UNIVERSITY OF CALIFORNIA, SANTA BARBARA

User Guide for AUTOTUNE

Open source repository

Latest version: https://github.com/yuyiyi/AUTOTUNE_GUIdevelopment

Revision history

September 2023	Initial upload
February 2024	Version update

Contact for inquiry

Ikuko Smith D.V.M. Ph.D.
ikukots@ucsb.edu

Training videos

The following demo videos are available:

Registration
Feature Detection
Spine Turnover
Input Mapping/Tuning Response (orientation tuning)
Input Mapping/Behavioral Response (locomotive behavior)

Demo datasets

The following demo data are provided to try out respective AUTOTUNE modules:

Demo image files

Stack-1: t-stack images for Registration and Feature Detection
Stack-2 & 3: t-stack images for Registration and Feature Detection

MATLAB bin files

Framestamp1: for Input Mapping/Tuning Response function of Input Mapping module to be used with ROI feature map MATLAB bin file (.roi) generated on Stack-1

Timepoint 1 & Timepoint 2: MATLAB bin file with ROI details for Stack-2 and 3; for Spine Turnover

Locomotive Behavior: MATLAB bin file for Behavioral Response function of Input Mapping module (contains framestamp and stampinfo)

1. Systems requirement

MATLAB R2019a or newer is recommended. In addition, the following MATLAB toolboxes are necessary to operate AUTOTUNE: Image Processing Toolbox, Signal Processing Toolbox, Curve Fitting Toolbox, Statistics and Machine Learning Toolbox, and Parallel Computing Toolbox. In order to achieve smooth data processing, operating systems equipped with sufficient Random Access Memory (RAM) and a multicore Central Processing Unit (CPU) are recommended. The software was developed and tested using 32 GB RAM and an Intel Core i7-5960X CPU with a clock speed of 3.0 GHz and a NVIDIA GeForce GTX 960 graphics card. Graphics Processing Unit (GPU) devices with computing capability above 5.2 is strongly recommended though not required (GPU 5.2 will not be supported by future version of MATLAB). Under these conditions, we observed that an image stack registration for motion correction could be performed at 22 GB/hr.

2. Software download

The latest version of the software, AUTOTUNE, can be freely accessed with a set of demo data from Github repository at https://github.com/yuyiyi/AUTOTUNE_GUIdevelopment. Users will download the software suite to the destination folder location of their choosing.

3. Starting AUTOTUNE

When a user starts MATLAB application, it is imperative to first add the location of the software folder in the MATLAB path. For this, simply find the downloaded software folder in the *Current Folder Window* to the left of the *Command Window,* right click on the folder to select “Add to path” and then “Selected folders and subfolders”.

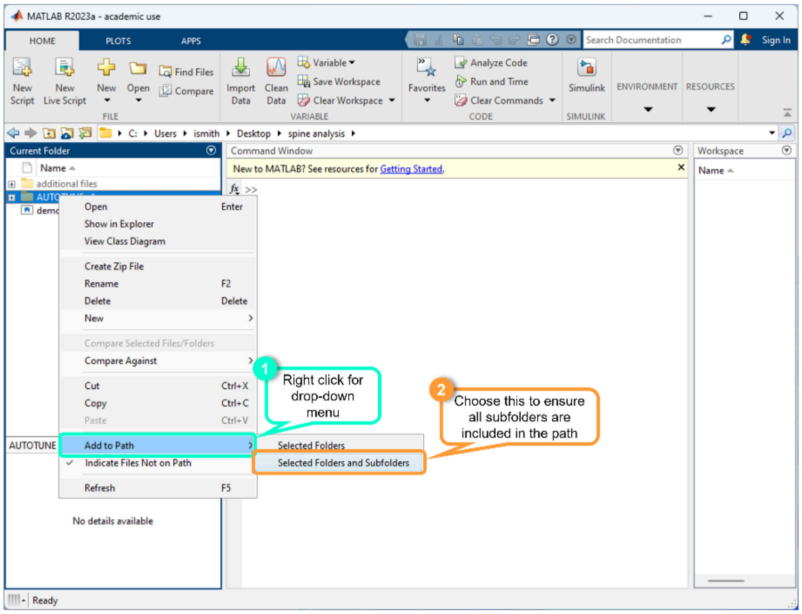

With the software folder successfully downloaded and added to the MATLAB path, enter “AUTOTUNE” (case sensitive) in the *Command Window* to start AUTOTUNE. The main control GUI will appear.

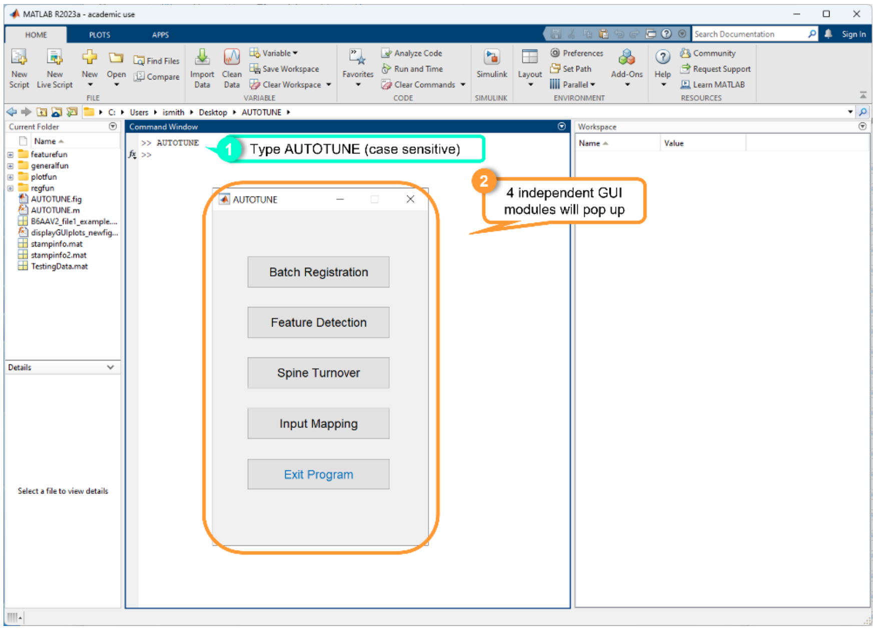

4. Batch Registration (motion correction)

Batch Registration module allows the users to register one or more 8-bit or 16-bit TIFF image sets. In order to perform image registration for motion correction of a set of individual movies or concatenate a collection of smaller movies into one, follow the steps below:

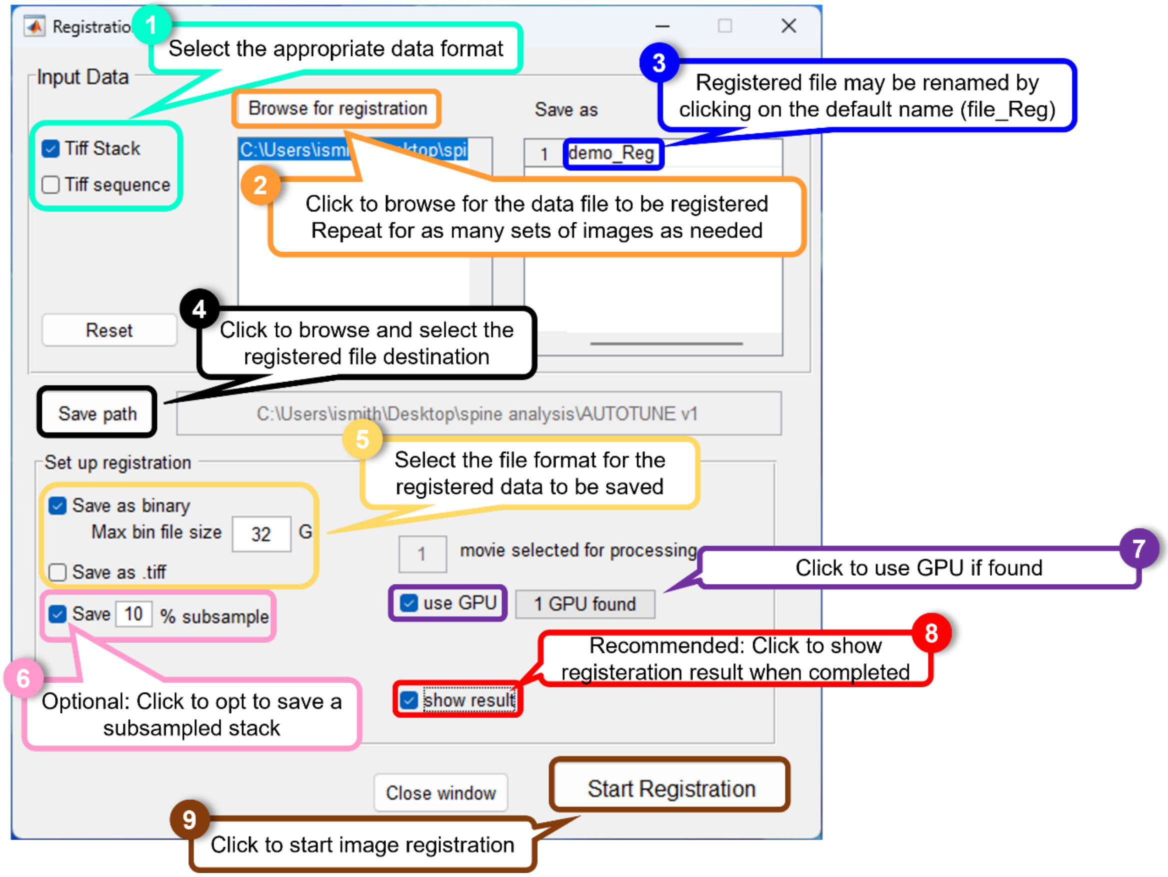

1. Select between 'TIFF Stack' and 'TIFF Sequence' for registering individual movies or concatenating a collection of movie stacks, respectively.
    a. When selecting TIFF stack option, users can input one or multiple TIFF stacks. And each TIFF stack would be registered and saved independently.
    b. When selecting TIFF sequence, a folder containing multiple TIFF images or multiple TIFF stacks would be concatenated and registered as a continuous recording. Users can input a super folder containing more than one subfolders of multi-TIFF stacks, in which case each subfolder would be registered independently.
2. Click *Browse for registration* to load image sets to be registered.
    a. Users are prompted to select TIFF stacks or folder of TIFF sequence, based on their selection from the previous step.
    b. Users can input data from different directories, by clicking the *Browse* button again to repeat the process. Movies are added to the *Browse for registration table* where it lists their respective directories. The table will not be refreshed until the module is closed or the *Reset* button is pressed.
3. Users can change the file name for the registration results if desired by clicking and retyping in the *Save as* table (SourceFileName_Reg).
4. Click *Save path* to locate the desired file destination for saving registration results.
    a. Default destination is the location of the last input source movie that was loaded.
    b. Reset button will clear all selected movies as well as the *Save Path*
5. Users must select between Binary or TIFF files for the data format of the registration results. **Binary files are strongly recommended as it significantly accelerates the overall registration and saving time.**
    a. For Binary files, users can either save the results in a small number of files with a large bin size or a larger number of files with a small bin size.
    b. The maximum size of a bin file is capped by the PC's computational power, and is automatically determined by AUTOTUNE and displayed in the Max bin file size box in the GUI.
    c. Users have the option to further decrease the file size by entering a smaller number in the box, but the input cannot exceed the initial cap calculated by AUTOTUNE.
    d. Saving results in smaller bin files is recommended as it would allow users to load/read the files faster on another computer with smaller RAM (e.g. laptops) or if a user wishes to load them in ImageJ.
6. It is optional to save a subsampled stack of registered images for a quick inspection later on.
7. Click to opt-in to use GPU. **It is strongly recommended as it significantly accelerates the process.**
8. Clicking *Show Result* button will allow users to view the registration result as "Pre" and "Post" z-projected image when it is completed.

a. Size of the registration result window can be manually adjusted by dragging a corner of the panel.

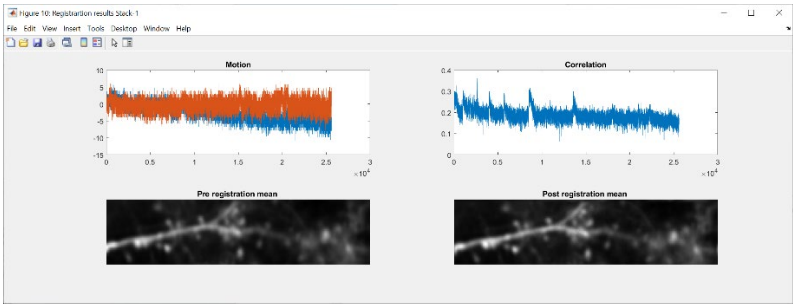

9. After all the settings have been selected, click *Start Registration*.
   a. A progress bar will appear to report the processing state, and a progress percentage (%).
   b. Elapsed time in seconds is tracked in the *Command Window*.
10. Close the module window after registration is completed.
11. The registration output is a registered movie in either *.bin or *.TIFF format and its corresponding *Parameter.mat file ("_RegParameter") which keeps registration metadata, pre and post registration t-projection, frame-by-frame xy-translations, and frame-by-frame registration correlation. All the outputs are stored in the save directory.
12. AUTOTUNE Registration module is designed to take into account the available memory of each machine and computes a proper batch size, avoiding any memory issues. However, it is important to note that the maximum number of frames in a single TIFF stack is capped at 2^{16}. Should the user generate a TIFF stack that exceeds this limit, split the stack into sequential substacks and use the "TIFF sequence" mode for batch registration.

5. Feature Detection

Feature Detection module allows the users to extract fluorescence-based activity transients from the four different subcellular compartments: dendritic spines, whole long dendrites, local dendritic shafts subregions evenly subdivided from the long dendrites, and local shaft subregions structurally coupled to ROI-defined spines. ROI detection can be performed both fully automatically and semiautomatically. For rigorous and thorough investigation of the segmented ROIs, augmenting with expert supervision and manual editing is highly recommended. Follow the steps below:

1. Select to indicate the format of the input source file.
   a. If working with a dataset that was registered using the AUTOTUNE *Batch Registration* module, users should select the option based on how the registered movie were saved from previous step (Binary or TIFF sequence).

b. If working with a new dataset, which was not registered with the AUTOTUNE, users will have the option to load in the TIFF stacks or TIFF Sequence, but not Binary files.
c. Binary files as input file can significantly accelerate the process and are highly recommended for a large stack or a sequence of images.

2. *Browse* to load the input source file.
    a. When opening registered movie saved as Binary files, select the "_RegParameter" file associated with the registration result.
    b. Binary files themselves are not visible when browsing.
    c. TIFF sequence files registered with the AUTOTUNE in the *Batch Registration* can be found in the "Processed" folder automatically created in the save file location set by the user.
    d. There, the TIFF sequence files are store in a subfolder ("_Reg").
3. Users can choose the save destination for the output file.
    a. Default destination is where the registered input source file is stored.

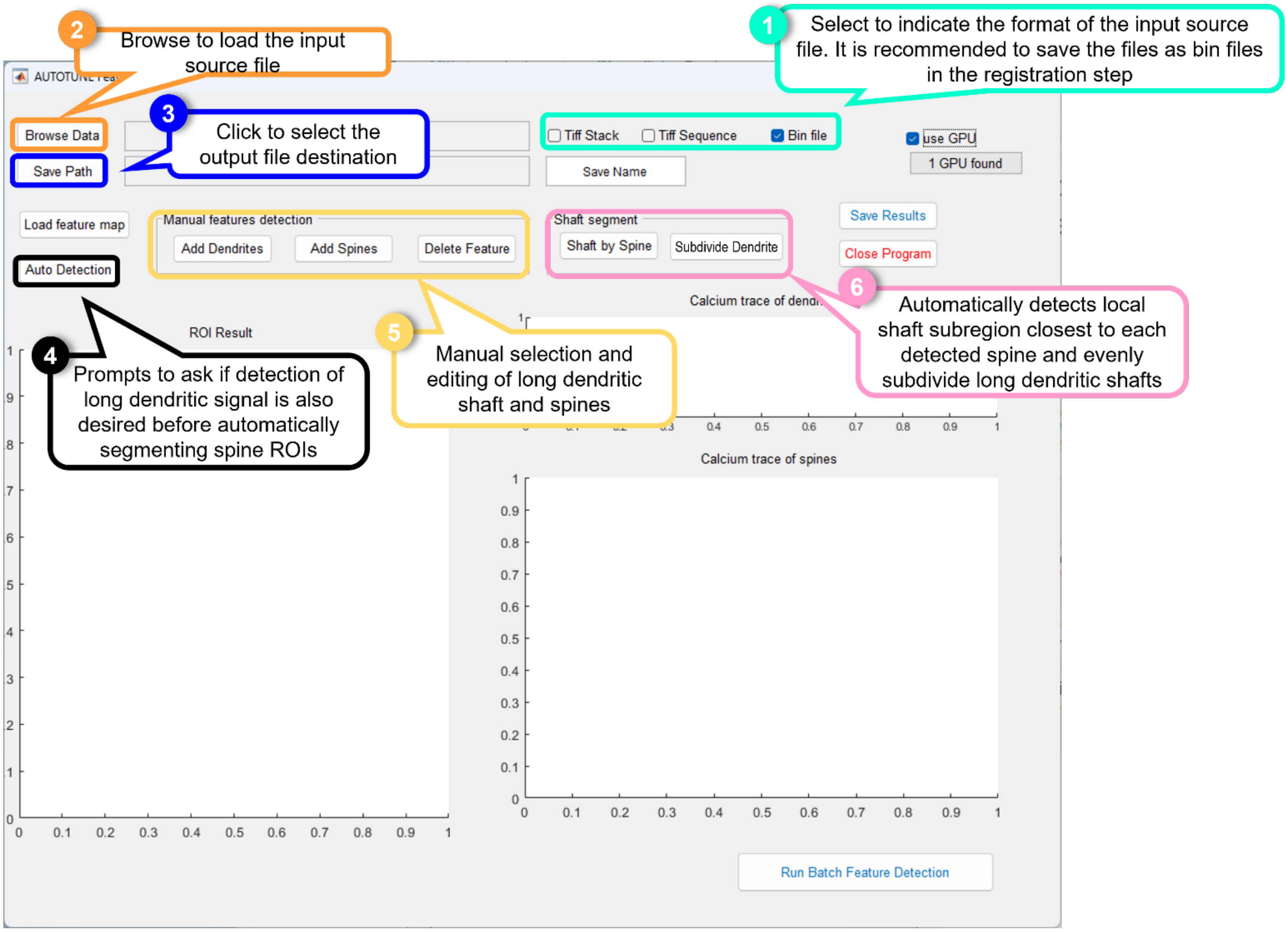

4. Auto Detection will prompt the user to choose whether they want to manually indicate the long dendritic shaft.

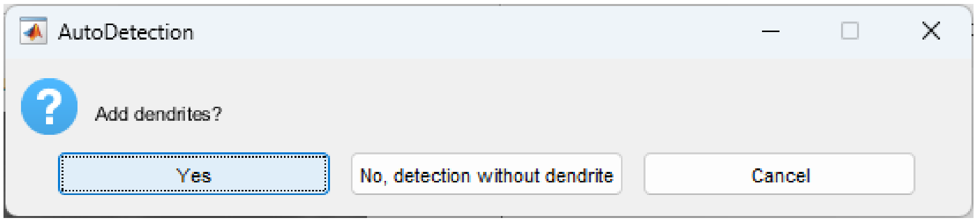

a. Selecting to indicate a dendritic shaft will automatically restrict the spine ROI detection to be those nearby the shaft.
b. Users can indicate as many dendrites as they wish. Simply select *Yes and Add* after accepting a shaft to move onto next. Make sure to select appropriate pixel size for *Set Line Width*.
c. Opting out of the manual dendritic shaft identification will prompt unrestricted small puncta detection regardless of spatial relationship to any dendritic branches.

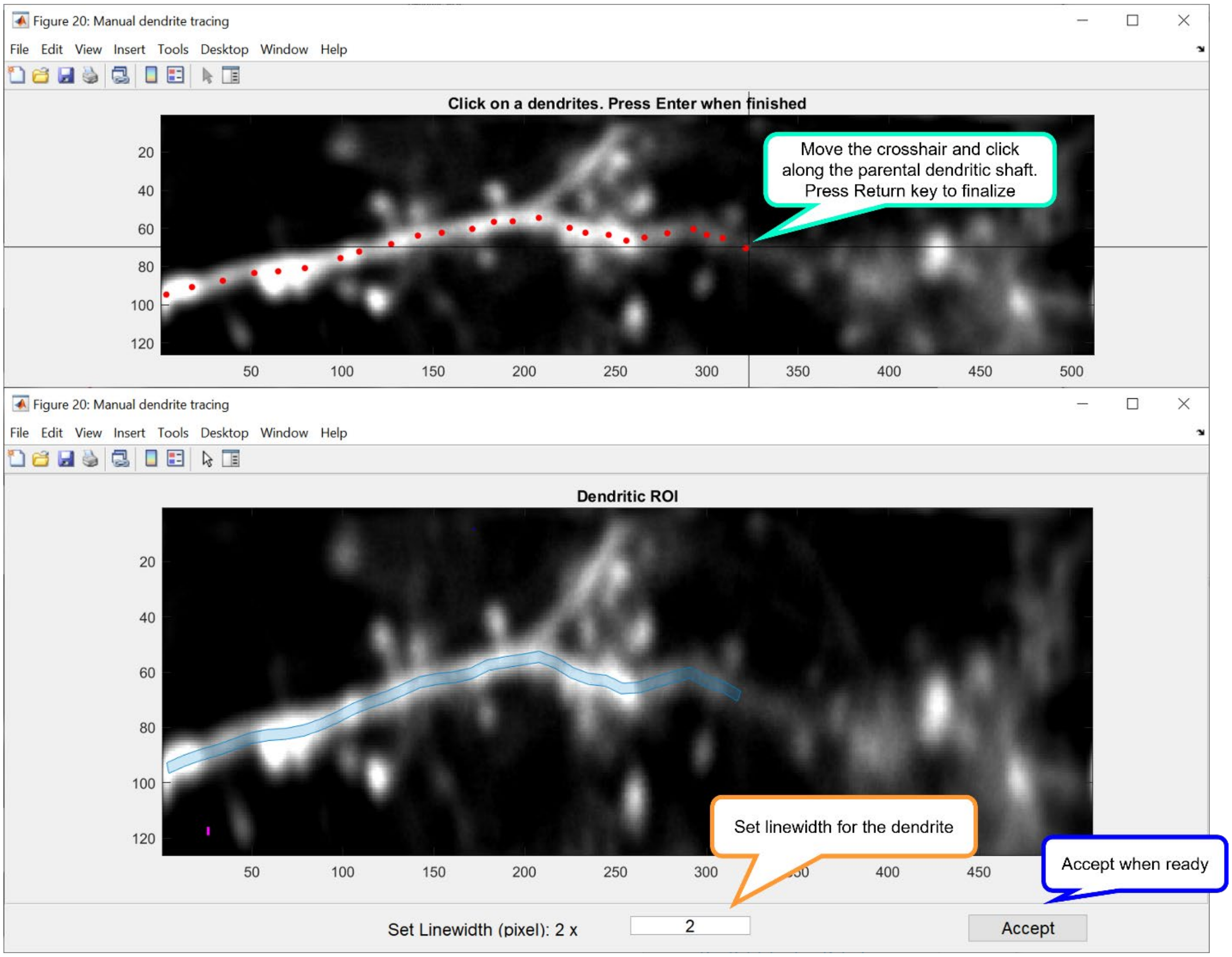

5. Users can also manually delete spines as well as dendrite ROIs. This step is recommended for cleaning up automatically detected ROIs.

a. *Add Spines* will open an average intensity heatmap of z-projected image, which will assist visualization of individual spines.
b. Users will click to indicate the centers of dendritic spines (ROI seeds).
c. Each click will display a segmented ROI and corresponding signals (e.g. calcium transients) in the *Spine Segmentation* pop-up window.
d. Press *Return key* on the keyboard to accept ROIs.

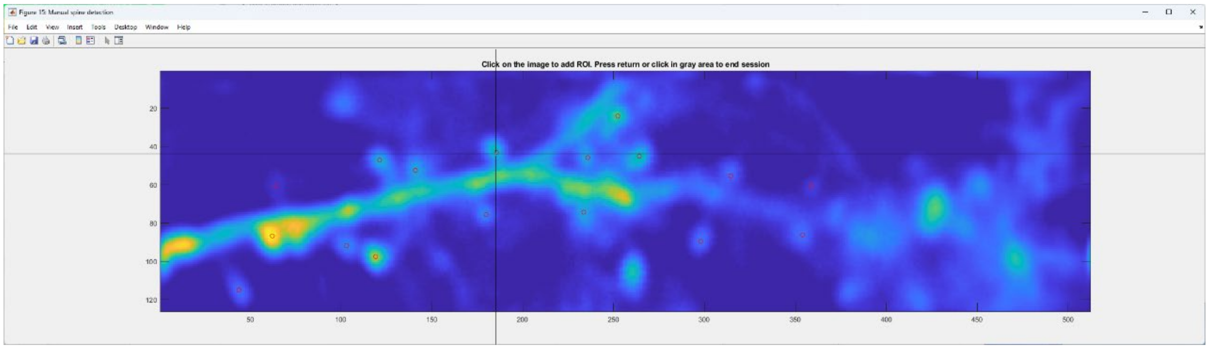

6. The module can also automatically detect local dendritic shaft subregion closest to each detected spine (left) or evenly subdivide long dendrites into small pieces (right).

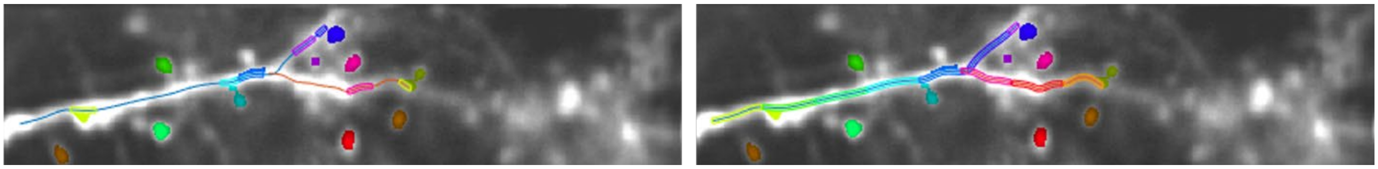

a. Users are prompted to set the length of the dendritic shaft subregion in pixels in a popup window.
b. Pixel value for the shaft width selected at Section 5.4b should serve as a reference in choosing the subregion width length.

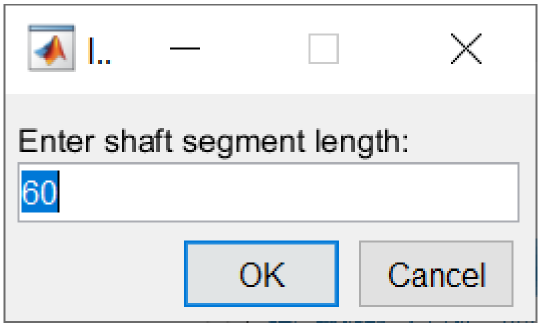

7. Press *Save Results*.
   a. Users can edit the name of the feature map by clicking and typing (default name ends in **roi.mat**).
   b. Default save destination is the same as the input source file.

8. Before finalizing the feature map, a popup window will appear, providing users an opportunity to specifically assign which long dendrite a given spine should be associated with.
    a. This process is useful especially when there are multiple dendrites in the image, allowing users to inspect whether AUTOTUNE assigned an appropriate dendrite to be associated with a given spine and reassign if needed.
    b. Default assignment of a dendrite is based on the proximity.
    c. In order to reassign a spine to different dendrite, the user must click on the spine ROI circle. In the even that a cursor is hidden, pressing *New* button will reactive the interactive figure.
    d. If no reassignment is needed, simply press *Finish*.

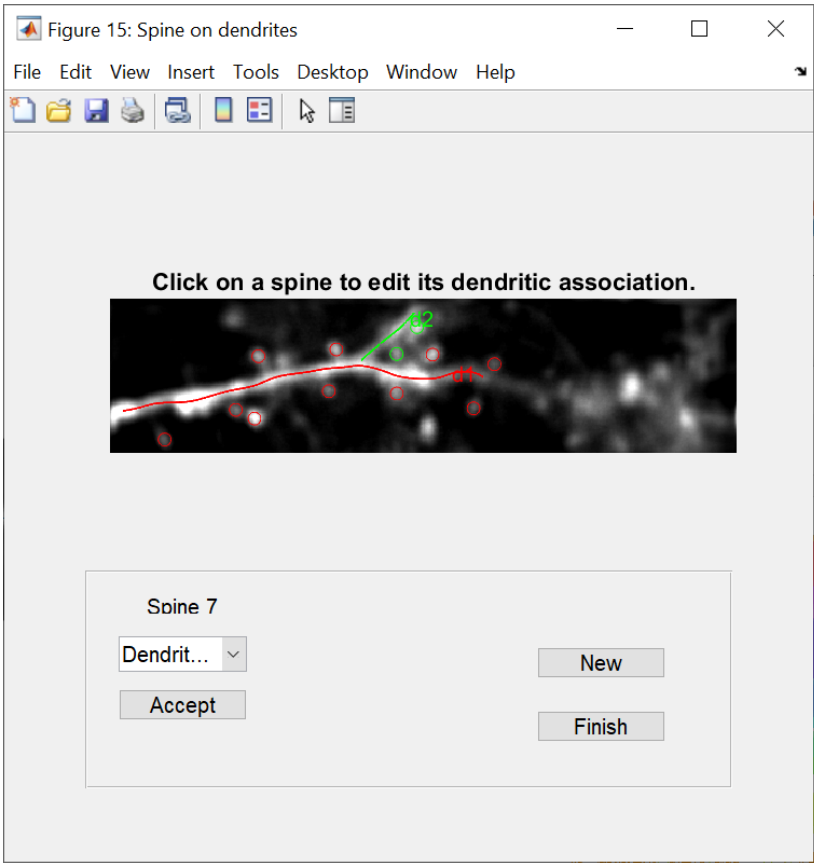

9. If a user has already gone through steps 1 through 7 in a prior session on a given image, *Load Feature Map* will apply an existing stored feature map onto a loaded movie.
    a. Users will be prompted to select a feature map.
    b. AUTOTUNE will first perform cross-session alignment before applying the mask onto the current selected registered movie.
10. Run *Batch Feature Detection* (also see Section 6 below)

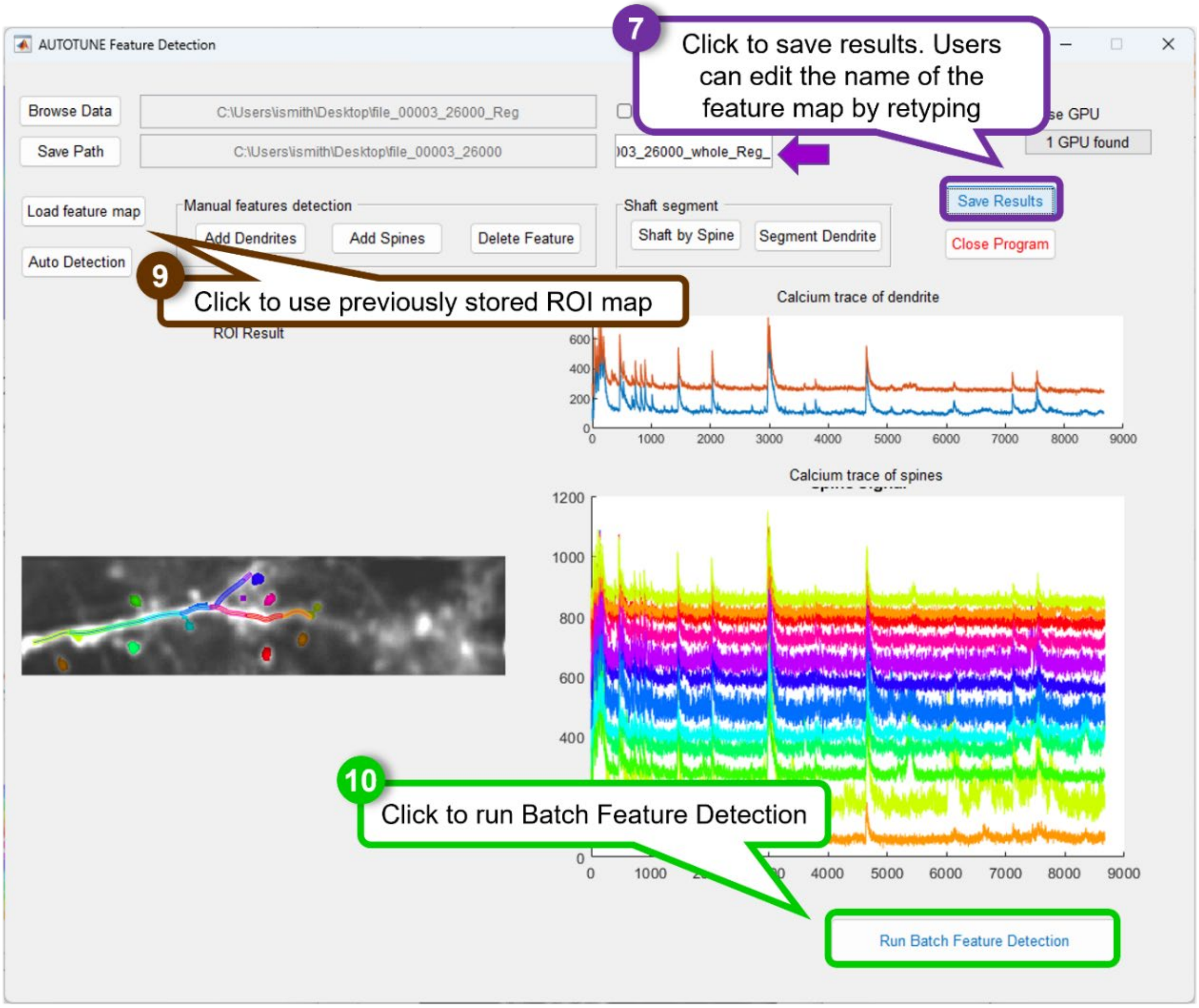

11. The *Feature Detection* outputs a *.mat file containing the following:
    a. an average image of the dataset (im_norm)
    b. dendritic shaft and spine features organized as dendriteROI (manually added by the user), spineROI (autodetection or manually picked by the user) and dend_shaft (dendritic subregions).
    c. Each of the ROI variables contains information about its pixel location (*_pixel), raw time series (*trace) and normalized trace (*dff).
    d. dff = (F-baseline)/baseline where baseline is defined as 40% quantile of the time series.

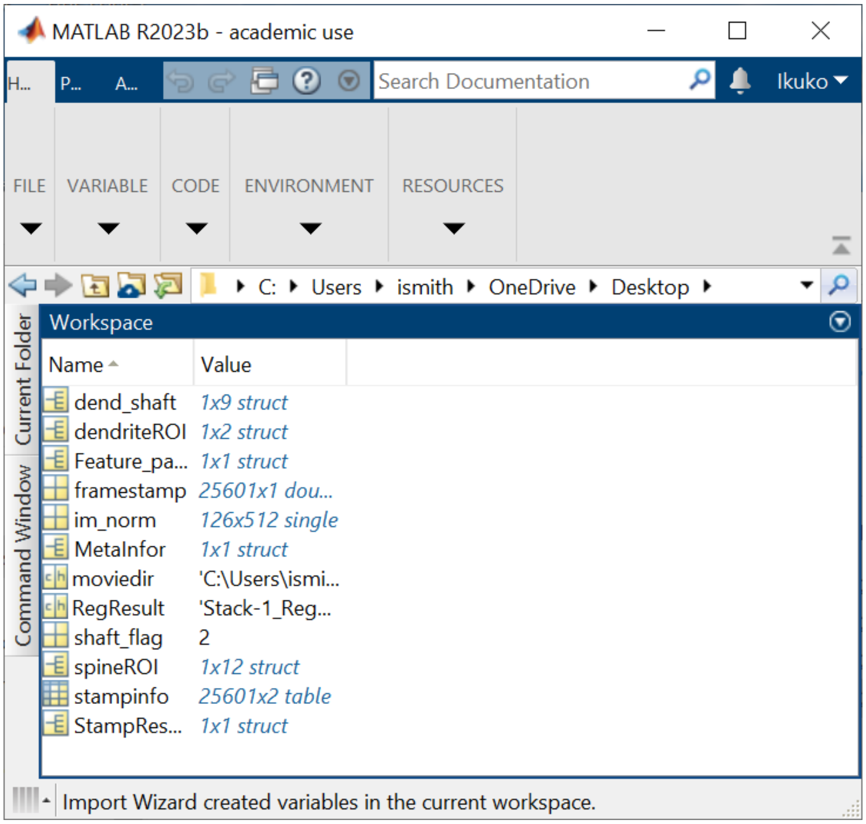

6. Batch Feature Detection

Batch Feature Detection module allows the users to apply a single previously generated common feature ROI map to multiple image sets taken from the same FOV across multiple sessions, times, and/or conditions. The module can also be used to batch process datasets that simply do not require manual feature detection ("same feature map applies all"). Functionally, it is the same process as *Load Feature Map* function (*section 5.8*) except it is executed in a batch. Follow the steps below:

1. Select to indicate the format of the input source file. Binary files are recommended for a speedy process.
2. Browse to load registered (i.e. motion-corrected) movies of interest in a batch.
3. Click *Batch processing with existing feature map* to select a previously generated common feature ROI map to be applied to all files (default name is SourceFileName_roi).
    a. If left unchecked, the module will simply run automatic feature detection on individual stacks separately without referencing an ROI map.
    b. It is recommended for optimal results but not required that the movies be motion-corrected.
4. With a Feature map loaded, click Run Batch to start the batch analysis in order to extract signals from the corresponding ROIs in all loaded files.

a. AUTOTUNE will first perform cross-session ICP image registration to align the new movie to the movie from the common feature map and then apply the common feature map onto the now realigned movie.

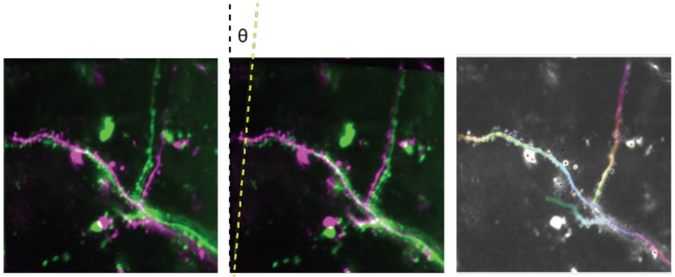

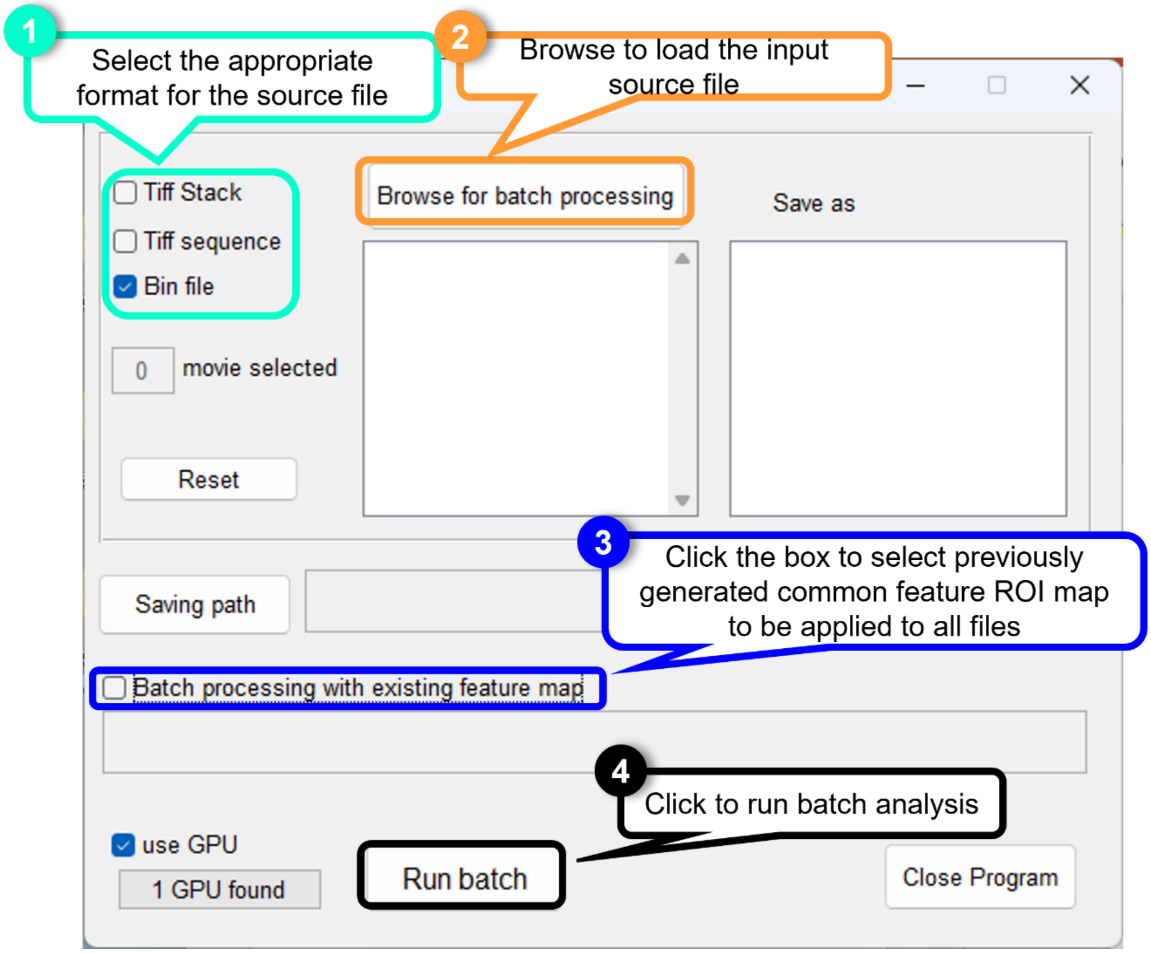

7. Spine Turnover

Spine Turnover module compares two or more saved feature maps and categorizes the spine ROIs of a selected target movie into lost, retained, or gained.

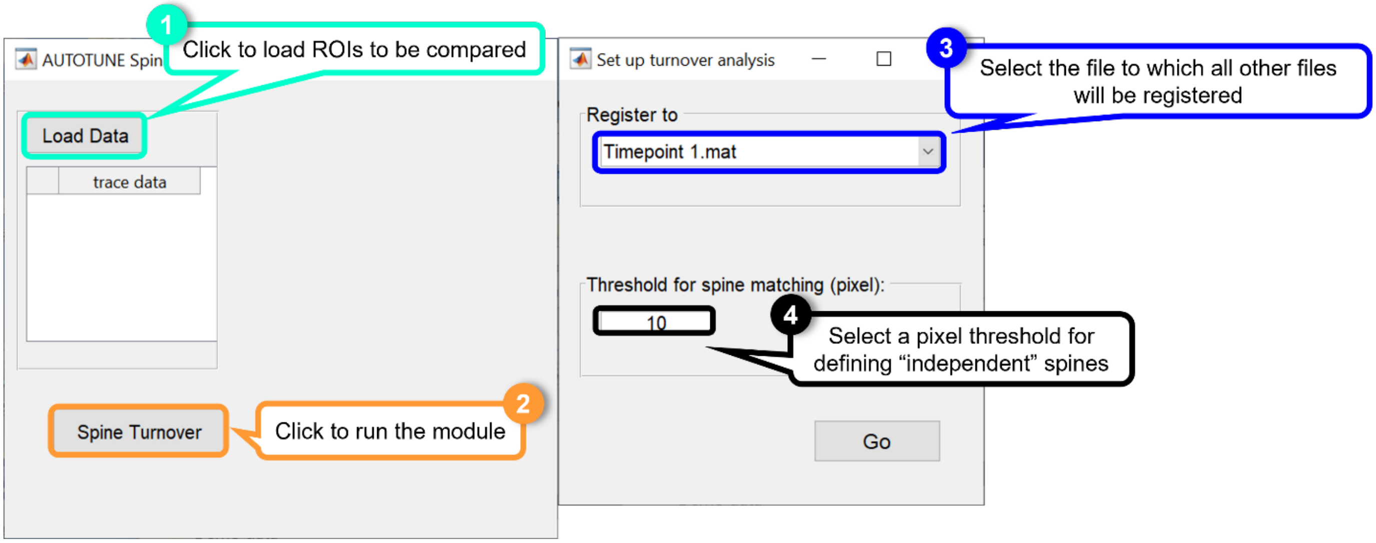

1. Browse to load multiple saved feature maps (ROI maps) that were generated by the AUTOTUNE *Feature Detection* module, to be compared.
2. Run Spine Turnover.
3. Select a target file to which all other files should be registered in a popup window.
    a. AUTOTUNE will perform cross-session alignment of each movie onto the target movie and then compare the associated feature maps.
4. Indicate a pixel threshold for defining how separated ROIs must be in order for them to be counted as different (and therefore categorized as lost or gained).
5. Spine Turnover results will show the following in separate popup windows:
    a. Registration results

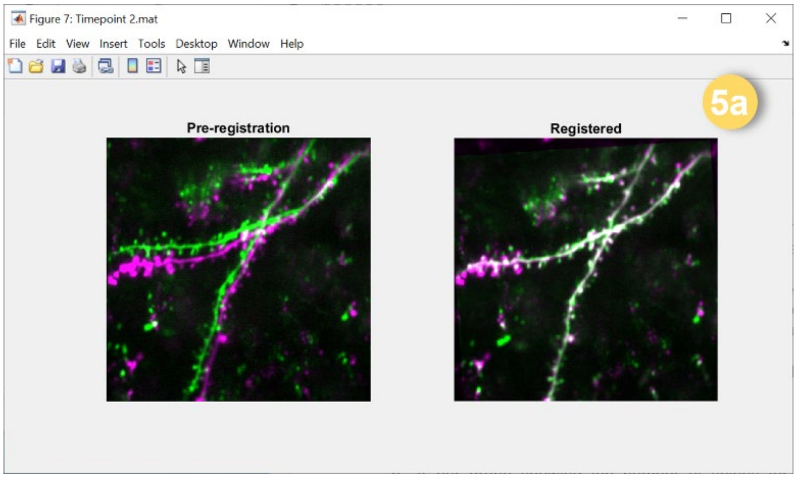

    b. Spine turnover results with each spines categorized and annotated as "lost" (green circle), "retained" (blue cross) or "gained" (red circle).
    c. A bar graph showing the number of spines for each category.
    d. A histogram showing spine ROI area distributions.
    e. A histogram showing the spine nearest-neighbor distance.

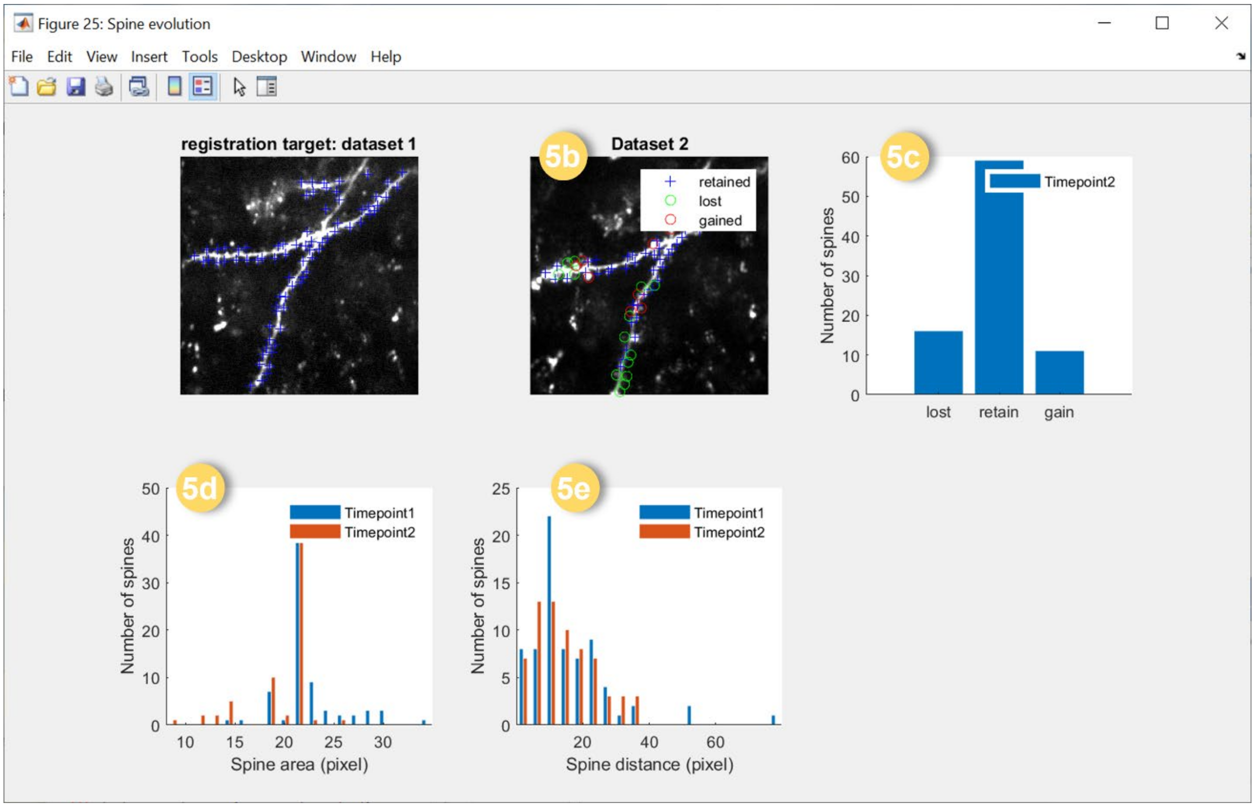

f. Spine locations along the associated dendrite.
   i. Toggle between dendrites and press *Go* to generate respective spine location graph on each dendrite if multiple dendrites exist within an image.
   ii. Select a pixel value for binning.

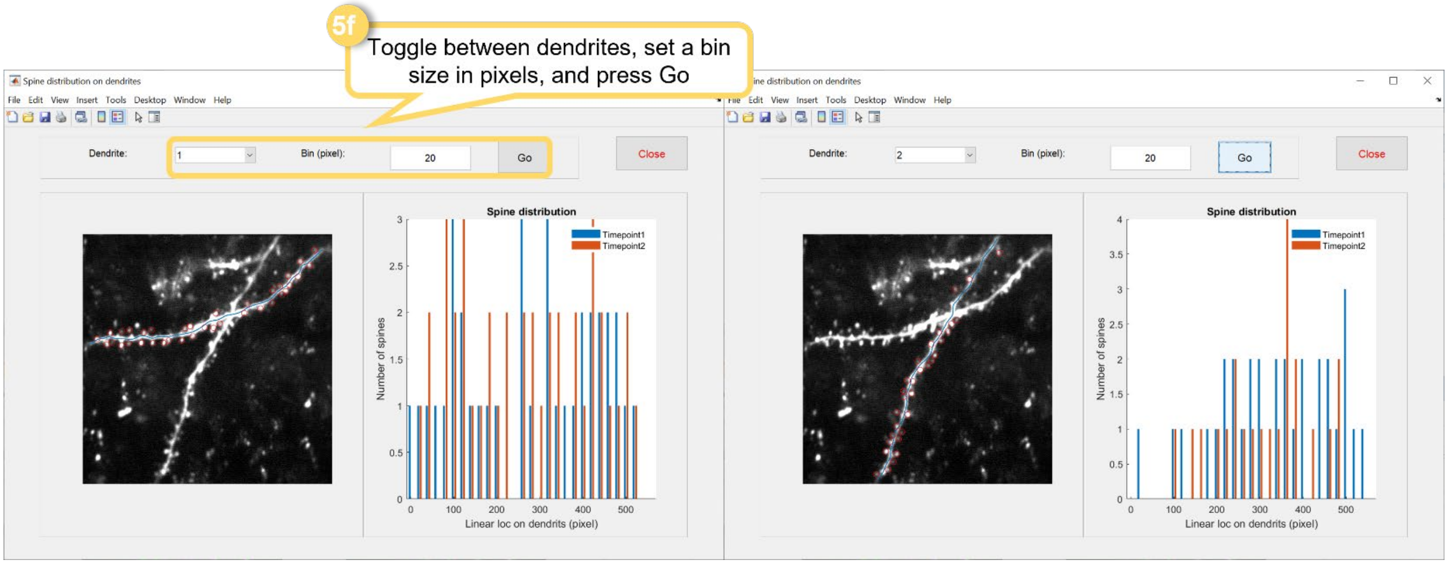

6. Spine Turnover results are saved in a new MAT-file with a prefix “SpineEvolveAnalysis_” and are organized into separate entries with associated contents:

Import	Name	Size	Bytes	Class
☑	spine_evolve	111x8	9791	table
☑	num_turnover	1x3	1443	table
☑	filelist	2x1	668	cell
☑	crossSessAlign_thresh	1x1	8	double
☑	crossSessAlign_target	1x1	132	cell
☑	TranM	1x2	736	struct
☑	Dendrite_CrossSess	3x4	1839	table

a. spine_evolve table: spine ID, spine area, linear location on dendrites and its associated dendrites. Spines are sorted by its matched spine ID in the target session.
b. num_turnover: number of lost, retained and gained spines for each session.
c. crossSessAlign_thresh: user-defined threshold pixel value used for spine turnover analysis
d. crossSessAlign_target: target file of the turnover analysis
e. filelist: file list of all cross-session data in the current analysis.
f. TransM: cross-session transformation matrix
g. Dendrite_CrossSess: cross-session alignment of dendrites

8. Input Mapping

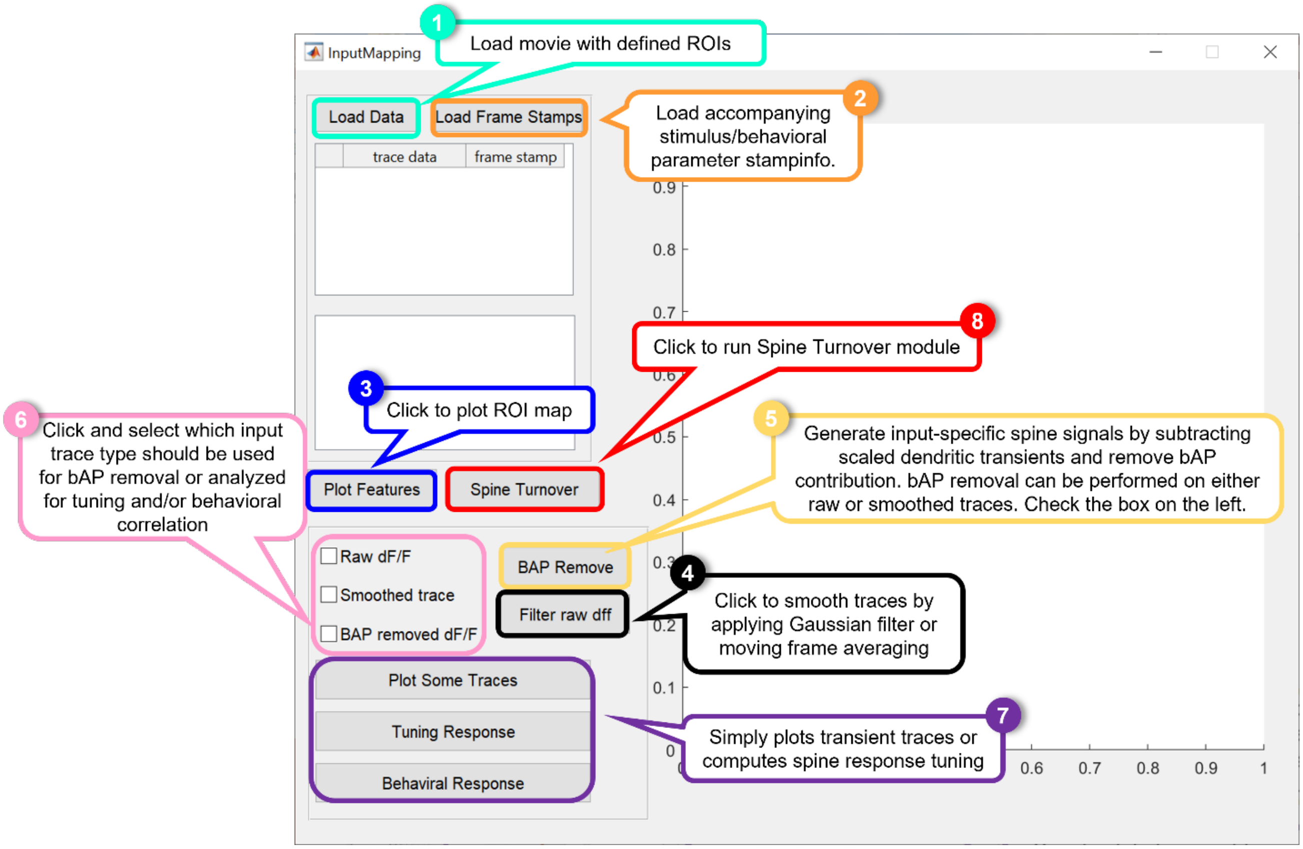

Input Mapping module is designed for examining response tuning properties of the dendritic spine activities relative to the stimulus or behavioral parameters. Conveniently, it can also be used to view the raw, bAP removed, and smoothed traces of as many ROIs as the user would like. Follow the steps below:

1. Click *Load Data* to input time series for features (ROI traces), which is either generated by the AUTOTUNE *Feature Detection* module (FileName_roi.mat by default) or a column matrix defined by the user.
    a. Multiple datasets can be loaded for batch analysis
2. Click *Load Frame Stamps* to select a *.mat file that contains the timekeeping information for the feature data ('framestamp') as well as temporally corresponding stimulus/behavioral parameter tables ('stampinfo') (e.g. orientations of the black and white moving gratings, locomotion speed, etc).
    a. *Load Frame Stamps* can overwrite the previously associated stamp files.
    b. Framestamp describes the time course vector of the imaging data (i.e. what each image frame is in whatever temporal unit is used in the stampinfo). As such, it should be the same length as the number of frames in the stack, or the number of timepoints in the activity transient trace.
    c. The default name of the timekeeping vector is 'framestamp'. If no 'framestamp' is found, users are prompted to pick a timekeeping vector in a different name in the loaded MAT-file.
    d. Stampinfo is formatted as a table containing the time courses of a set of stimulus/behavioral parameters.
    e. First column of the stampinfo table must contain timekeeping information for the stimulus/behavior parameters. The rest of the columns would contain information about stimulus properties (e.g. orientation of gratings), behavioral parameters (e.g. locomotion speed, animal position, etc), and trial information (e.g. closeloop vs openloop).
    f. The default name of this table is 'stampinfo'. If no 'stampinfo' is found, users are prompted to pick a table in a different name in the target MAT-file.
    g. The timekeeping information for features (ROIs; framestamp) and stimulus/behavior (stampinfo) should be expressed in the same unit, but can have different sampling rate.
    h. See the main text for more information on the data formats for the framestamp and stampinfo files.
    i. framestamp and stampinfo will be saved in the *mat file the first time they are loaded with a feature map and will automatically appear under *frame stamp* column.
    j. The inputted feature dataset and its corresponding stimulus/behavior information will be displayed in the *trace data* table.
    k. Clicking on each dataset in the *trace data* table allows for the visualization of its features.
3. *Plot Features* will allow the users to visually inspect the feature ROIs of selected dataset. Users can select a dataset in the *trace data* table.

4. Users may opt to smooth the extracted ROI transients by applying Gaussian filter or moving frame averaging.
    a. Users can input the following smoothing parameters in a popup window.
        i. Window size (frames) for smoothing by moving average
        ii. Frame rate (Hz), signal filter size (seconds), and baseline filter size (seconds) for denoising by Gaussian filter.

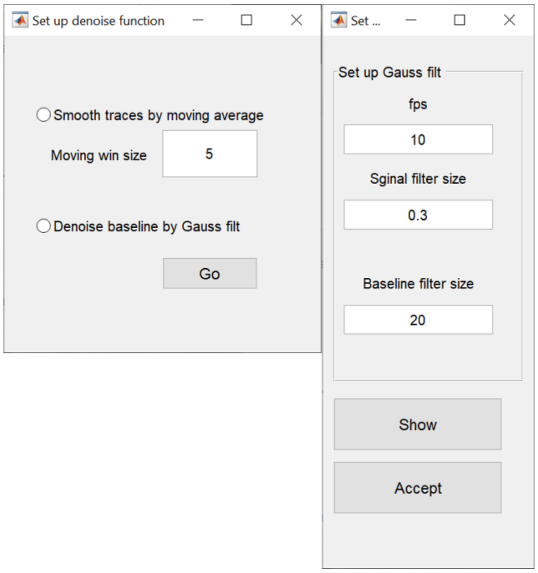

    b. Smoothed traces will be saved automatically in the original input feature data MAT-file. Named as *dff_filt.
5. Executing *BAP Remove* will start the process for automatic bAP removal, and opens a window asking if the user would like to manually perform bAP subtraction.
    a. If *no*, the module will run the bAP removal automatically without user supervision.
    b. If *yes*, the “robustfit” plot between the activities of a dendritic shaft and individual spines and shaft subregions will be displayed to aid the user with adjusting the scaling factor (see *3.6.2 Removal of back-propagating axonal action potential signals* in main text).
    c. When multiple dendritic shafts exist in the ROI map, the one that was assigned to each spine during *Feature Detection* will be automatically used.
    d. As they inspect the individual “bAP removed” traces, users can use the sliding bar to finely adjust the scaling factor (slope) for the bAP removal so as not to introduce any artificial negative deflection caused by over-subtraction. Press accept when satisfied with the resultant trace for each spine.
    e. The bAP removed traces, bAP removal coefficients (scale factor) and the scaled dendrite trace (carrying estimated bAP contribution to the spine and local shaft subregion signals) are saved automatically to the original feature data MAT-file.

f. Users have the option to perform bAP removal on raw dF/F traces or smoothed traces by checking the respective box on the left.

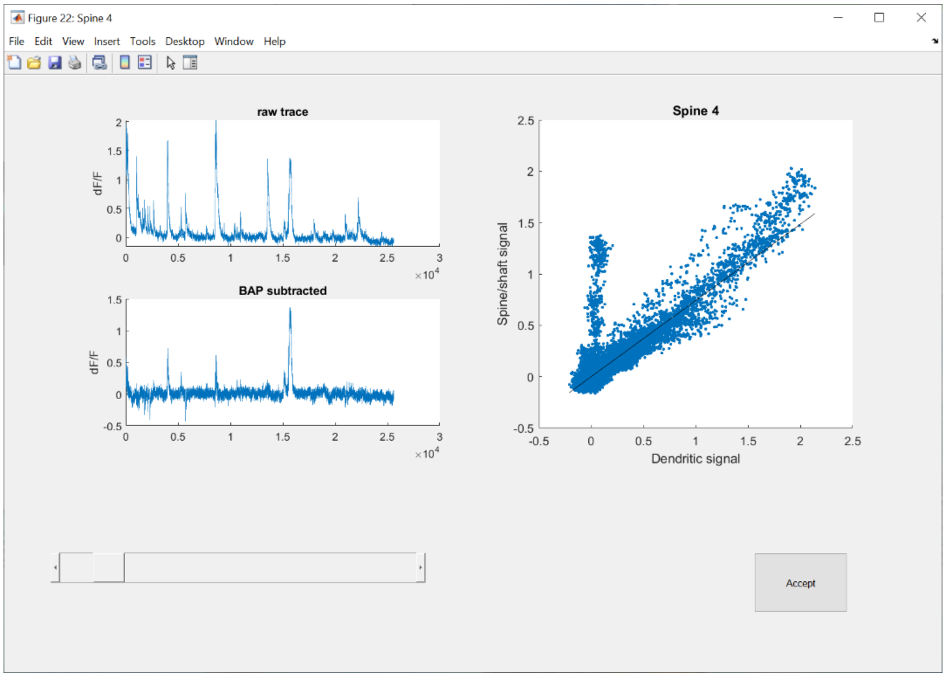

6. Users have the option of using Raw dF/F, Smoothed dF/F, and/or bAP removed dF/F traces for the subsequent *Plot Traces, Tuning Response* and *Behavioral Response* functions.
7. *Plot Traces* will generate the extracted transient traces for the selected ROIs, showing 10 at a time which the user can advance through by clicking on the figure window.
   Tuning Response will generate a tuning curve of the transients relative to the stimulus/behavioral features for each ROI selected by averaging its response during each stimulus window. When promped, select the stimulus/behavioral variable against which a tuning curve should be plotted.

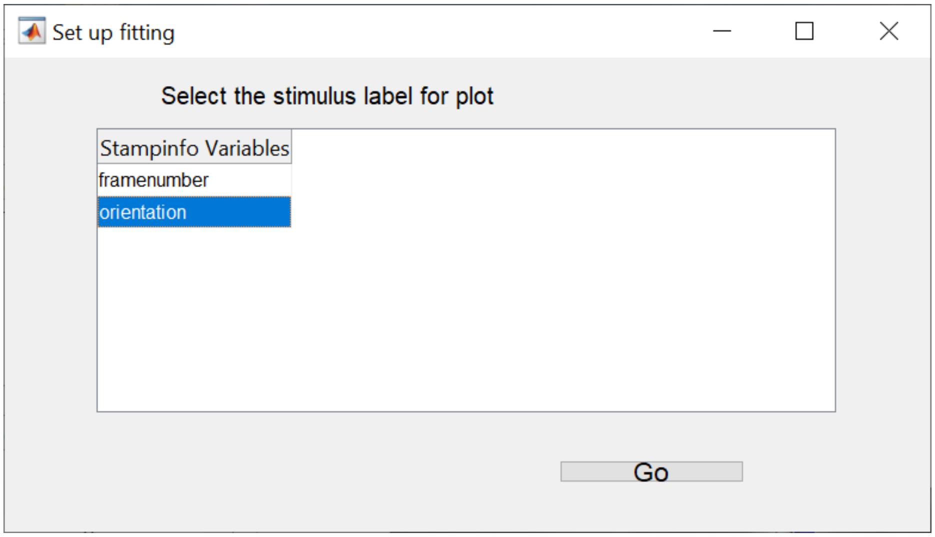

Simple fitting (line,

Gaussian, sigmoidal, or quadratic) as well as custom user-defined fitting options are provided. If set to *none*, the program computes the average response and standard error during each stimulus window.

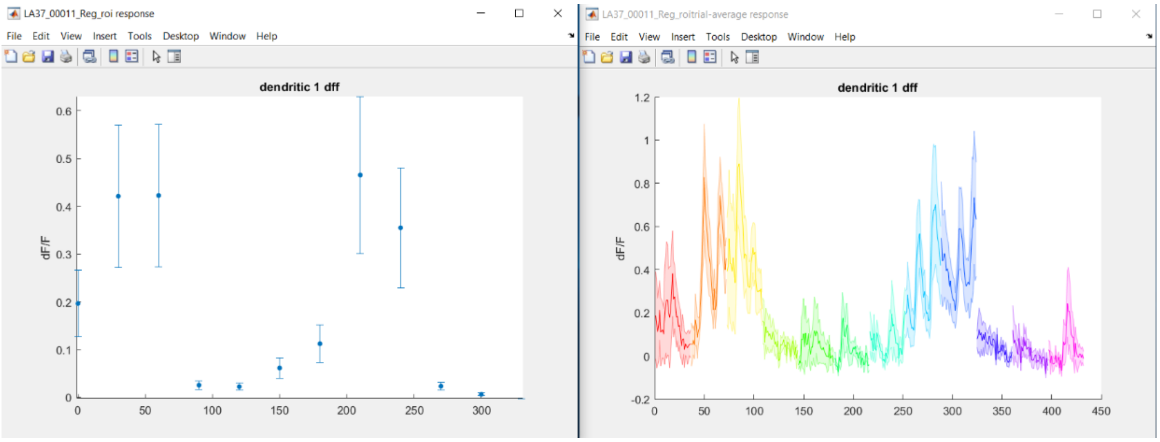

a. Two-peak Gaussian fitting is included specifically for the orientation tuning analyses of visually responsive dendrites. In the equation, the peaks are constrained to be apart by π. For circular Gaussian fitting (one-peak or two-peak), users must provide radius for the stimuli.
b. Users can select all or a subset of stimuli label for tuning analysis. For example, one may exclude the gray period in between orientated-bar stimuli (for simplicity, 45-degree increment orientations from 0 to 315 degrees are expressed as 1-8 while gray period is indicated as 0 in this example).

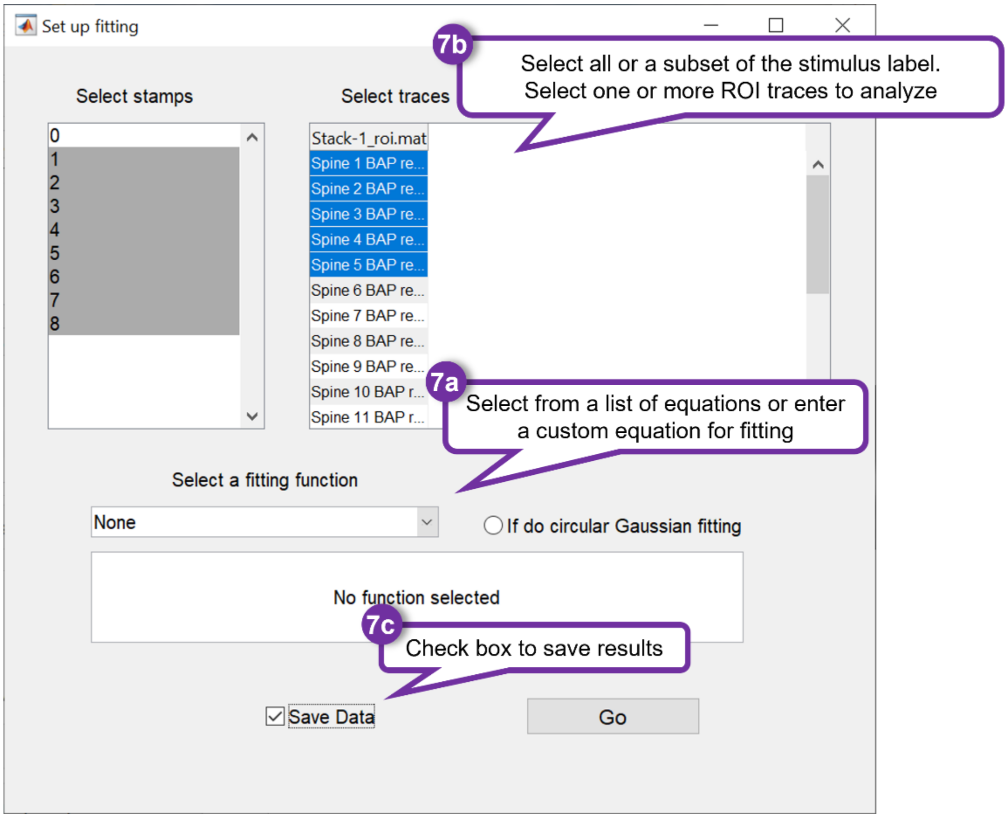

c. Checking *Save Data* box will save the results of the tuning analysis in the original feature data MAT-file, with a default prefix ‘Stamp_Resp_*’.

Behavioral Response allows the users to perform refined analyses with multiple variables, closely examining potential correlative relationships between dendritic spine responses with complex experimental parameters such as behavioral parameters with or without stereotyped trial structure (e.g. duration, condition, etc).

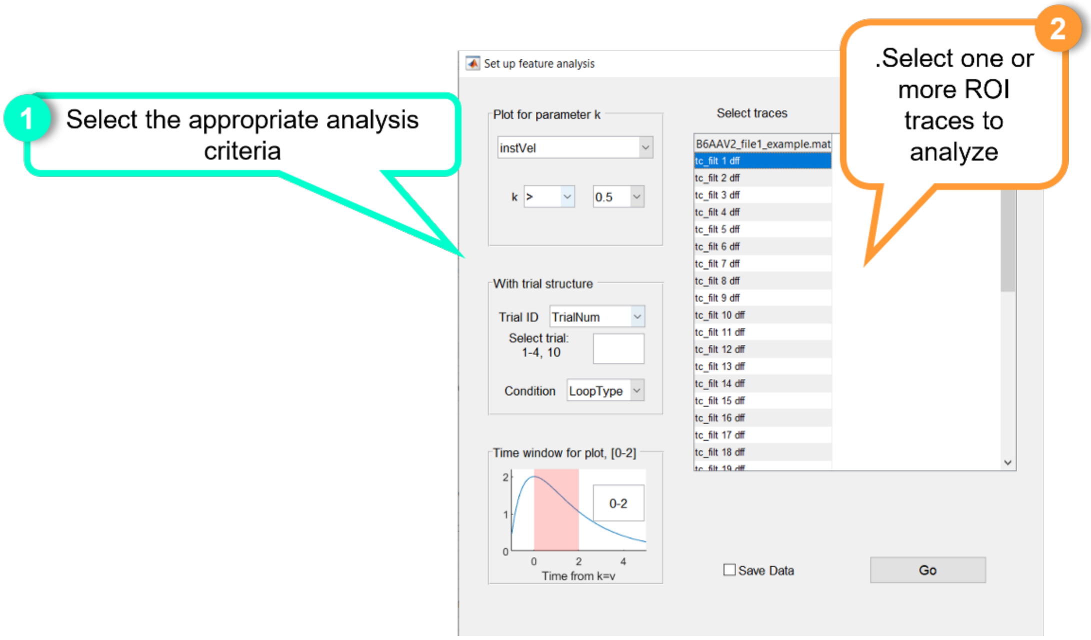

a. Use the drop-down menu under *Plot for parameter K* and select the parameter to inspect and set a threshold value (k =, >, or <) to generate an event-triggered average for selected traces.

d. *Within trial structure* allows the users to select additional behavioral parameters or conditions as constraints for computing event-triggered average. The drop-down menu will list the variables in the stampinfo containing trial identity and trial condition labels. Users can also define the range of trial identity to analyze (*Select trial*:)

e. *Time window for plot* allows the users to define a window for event-triggered averaging.

f. The program will generate plots of event-triggered average response (Mean +/- SEM) of selected features and for each condition (for a user-defined time window with same amount of duration ahead of the event. Time 0 indicate the event initial.

g. Locomotion demo dataset plots the instant velocity of animal locomotion (instVel in stampinfo table) with the following set criteria with using the parameters from the stampinfo table:

    i. Parameter k = instVel; k>0.5 (cm/s)
    ii. Time window = 2 s
    iii. Conditions; closeloop (LoopTye = 1) vs openloop (LoopTeyp = 2)

iv. Red = condition 1; Blue = condition 2

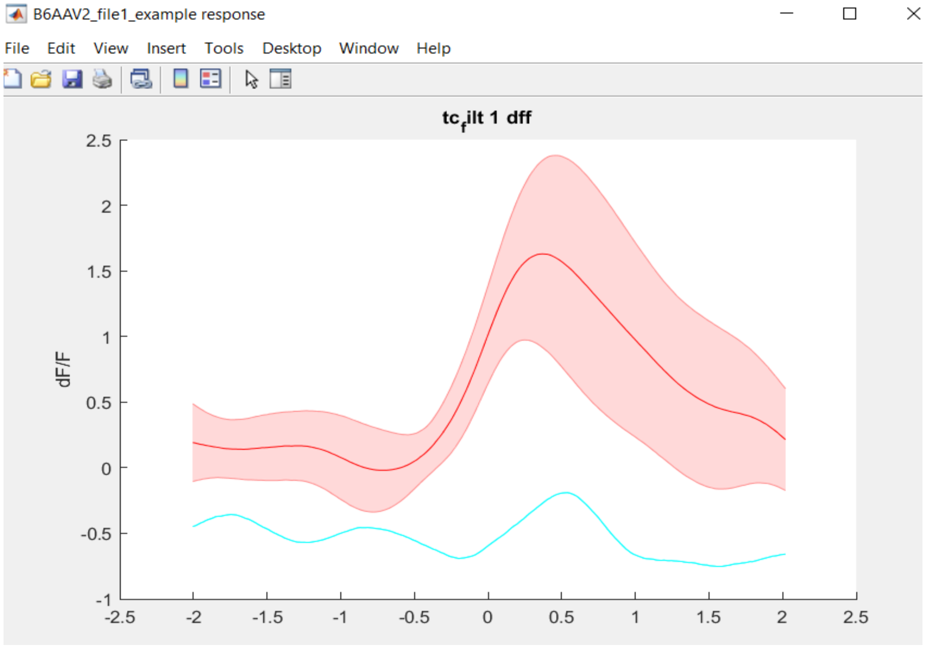

h. Check Save data box to save the event-triggered tuning analysis results in the original feature data MAT-file, with a default name 'BehavResp*'.

8. *Spine turnover* button will perform the same analysis as the *Spine Turnover* module on all selected datasets.

9. Editable default parameter list (util/defaultparameter.m)

Users can edit the default parameters listed below by following the commented instruction in the file.

```
% ----------------- edit with caution -------------------------------
%% movie denoise for feature detection (Don't change these parameters unless you are confident)
para_default.GaussKernel = [4, 4, 2];   % movie denoise kernel
para_default.maxLength = 4000;          % default dendritic width (pixel)

%% parameter to feature segmentation
para_default.linewidth = 6;             % default dendritic width (pixel) (popup variable in GUI)
para_default.th_grad = 2;               % feature segmentation at 1/th_grad of the correlation map
                                        % increase this value would generate larger segmentation,
                                        % change range > 1
para_default.w = 3;                     % feature segmentation neighborhood = w*linewidth
                                        % increase the value, correlation map would be computed from a larger neighborhood

%% parameter to auto feature detection
para_default.minarea = 5;               % minimal allowed feature size (pixel)
para_default.maxareagrad = 4;           % maximal allowed feature area (pixel) = linewidth*maxareagrad
para_default.MaxAR = 4;                 % maximal allowed feature aspect ratio
para_default.autofeature = [2, 3.5];    % parameter for autofeature detection (don't change unless you are confident)
para_default.autofeature_bg = 0.3;      % background thresholding parameter
                                        % below this fraction of intensity
                                        % pixel will be counted as
                                        % background if not manually
                                        % selecting background

para_default.ifbg = 0;                  % if manually select background for auto detection
                                        % set to 1, a window would pupup
                                        % allow users to select a background
                                        % area during autofeature detection. Facilitate
                                        % autodetection

para_default.shaftlength = 60;          % default shaft length in pixel (popup variable in GUI)
para_default.spinedist = 3;             % distance from dendrites for autodetection
                                        % spine search: spinedist * linewidth

%% cross-session alignment (Don't change these parameters unless you are confident)
para_default.ops.withrotation = 1;      % alignment with rotation 0(without)/1(with)
para_default.ops.maxIter = 150;         % max iteration
para_default.ops.tot = 10^-4;           % max tolerance
para_default.ops.distTh = [2, 50];      % [min max] distance threshold
para_default.ops.dispreg = 0;           % display
para_default.ops.pointsdetection = [2, 2.5, 2]; % point cloud detection parameters

%% motion correction (Don't change these parameters unless you are confident)
para_default.RegPara.PhaseCorrelation  = 1;     % set to 0 for non-whitened cross-correlation
para_default.RegPara.SubPixel = Inf;            % 2 is alignment by 0.5 pixel, Inf is the exact number from phase correlation
para_default.RegPara.maxDispPerFrame  = [];     % maximal displacement per frame. recommend [20, 20];
                                                % If empty, values set by 3.5*SD of displacement at initial alignment
                                                % Smaller value is not recommended

para_default.RegPara.lowCorr = 0.1;             % minimal registered-to-target correlation. Larger value is not recommended

% initialize motion correction
para_default.RegPara.NiterPrealign = 20;        % Number of iteration for initial registrition
para_default.RegPara.iniSearchiter = 5;         % Max iteration for chunk searching for initial registrition.
para_default.RegPara.FrameNoiniAlign  = 100;    % Number of frames for initial alignment.
                                                % This value would be bounded by system memory
                                                % and video length in processing
para_default.RegPara.MinCorr_initial = 0.2;     % chunk search continue when initial registrition correlation below this value

%% denoise trace (Input Mapping and Spine Turnover module)
para_default.Denoise.movingaverage = 5;         % moving average window size
para_default.Denoise.gaussfilt = [10, 0.3, 20]; % gaussian filtering [fps, signal filter kernel, baseline filter kernel]
para_default.spineRetain = 10;                  % threshold for mapping cross-session spines
```
